# Innate gene repression associated with *Mycobacterium bovis *infection in cattle: toward a gene signature of disease

**DOI:** 10.1186/1471-2164-8-400

**Published:** 2007-10-31

**Authors:** Kieran G Meade, Eamonn Gormley, Mairéad B Doyle, Tara Fitzsimons, Cliona O'Farrelly, Eamon Costello, Joseph Keane, Yingdong Zhao, David E MacHugh

**Affiliations:** 1 Education and Research Centre, St. Vincent's University Hospital, Dublin 4, Ireland; 2 Conway Institute for Biomolecular and Biomedical Research, University College Dublin, Dublin 4, Ireland; 3 Tuberculosis Diagnostics and Immunology Research Centre, School of Agriculture, Food Science and Veterinary Medicine, College of Life Sciences, University College Dublin, Dublin 4, Ireland; 4 Central Veterinary Research Laboratory, Backweston. Co. Dublin, Dublin, Ireland; 5 School of Medicine, Trinity College, St. James's Hospital, Dublin 8, Ireland; 6 Computational and Systems Biology Group, Biometric Research Branch, National Cancer Institute, Rockville, Maryland, USA; 7 Animal Genomics Laboratory, School of Agriculture, Food Science and Veterinary Medicine, College of Life Sciences, University College Dublin, Dublin 4, Ireland

## Abstract

**Background:**

Bovine tuberculosis is an enduring disease of cattle that has significant repercussions for human health. The advent of high-throughput functional genomics technologies has facilitated large-scale analyses of the immune response to this disease that may ultimately lead to novel diagnostics and therapeutic targets. Analysis of mRNA abundance in peripheral blood mononuclear cells (PBMC) from six *Mycobacterium bovis *infected cattle and six non-infected controls was performed. A targeted immunospecific bovine cDNA microarray with duplicated spot features representing 1,391 genes was used to test the hypothesis that a distinct gene expression profile may exist in *M. bovis *infected animals *in vivo*.

**Results:**

In total, 378 gene features were differentially expressed at the *P *≤ 0.05 level in bovine tuberculosis (BTB)-infected and control animals, of which 244 were expressed at lower levels (65%) in the infected group. Lower relative expression of key innate immune genes, including the Toll-like receptor 2 (*TLR2*) and *TLR4 *genes, lack of differential expression of indicator adaptive immune gene transcripts (*IFNG, IL2, IL4*), and lower *BOLA *major histocompatibility complex – class I (*BOLA*) and class II (*BOLA-DRA*) gene expression was consistent with innate immune gene repression in the BTB-infected animals. Supervised hierarchical cluster analysis and class prediction validation identified a panel of 15 genes predictive of disease status and selected gene transcripts were validated (*n *= 8 per group) by real time quantitative reverse transcription PCR.

**Conclusion:**

These results suggest that large-scale expression profiling can identify gene signatures of disease in peripheral blood that can be used to classify animals on the basis of *in vivo *infection, in the absence of exogenous antigenic stimulation.

## Background

*Mycobacterium bovis *infection is the cause of bovine tuberculosis, an important health problem in cattle with zoonotic potential for transmission to humans. In cattle this infection can be slowly progressive, with limited outward signs of disease, making diagnosis and eradication of tuberculosis difficult. Current diagnostic techniques often involve an *in vivo *single intradermal comparative tuberculin test (SICTT), alone, or combined with an *in vitro *ELISA based interferon-γ assay (IFN-γ) [1,2]. However, problems remain with the sensitivity of current diagnostics leading to a failure to detect all infected animals [3,4].

Following initial exposure to *M. bovis*, a specific T-cell immune response develops characterized by the release of proinflammatory cytokines including IFN-γ [5,6]. The loss of this early proinflammatory cytotoxic response is thought to be associated with an inability to control infection, resulting in progression to clinical disease [6,7]. The persistence of infection leading to chronic tuberculosis may be due to an ineffective immune response that involves suppression of specific immune mechanisms [6].

The immune response to tuberculosis is a complex process and studies in the bovine model have primarily focused on the adaptive immune response. Although the T-cell response is critical in controlling tuberculosis infection in cattle [6], studies in mice and humans suggest a significant role for innate immune mechanisms in mounting early and effective immune responses to mycobacterial infection [8-10]. Development of an effective adaptive immune response is dependent on innate immune activation. The innate immune response is regulated via receptors for antigen recognition known as pathogen recognition receptors (PRRs) and antigen presentation molecules. PRRs including the Toll-like receptors (TLRs) have been implicated in the immune response to *M. bovis *BCG [11,12], specifically TLR-2 and TLR-4 [9]. A diverse range of mechanisms used by mycobacteria to subvert the host immune response have also been described [13,14]. Mycobacteria can inhibit host cell signalling via the TLRs and other mediators of the innate immune response [15]; they may also interfere with maturation of the phagosome in infected macrophages, thereby reducing the ability of the host to successfully eliminate the pathogen [16,15]. Failure or subversion of an appropriate innate immune response may therefore be critical to the establishment of infection and progression to disease [6].

In recent years, high-throughput genomic analyses have facilitated identification of transcriptional regulatory networks involved in the orchestration of the immune response [17,18]. Gene expression studies of host responses to infection can provide a powerful tool for understanding the interactions between pathogens and the host immune system and may be particularly powerful in identifying specific molecules or pathways that have been targeted by pathogens for immune evasion [19]. One desirable outcome of genomic analyses across large gene subsets is the identification of an infection expression signature that may be used to differentiate groups based on their infection status [20]. Microarrays have recently been applied to the study of *M. tuberculosis *infection [18] and unique host gene expression signatures have been attributed to specific strains of *M. avium *in human macrophages [21]. In cattle, microarray studies of peripheral blood mononuclear cells (PBMC) from *M. avium *subsp. *paratuberculosis *(MAP)-infected cattle have revealed MAP-associated gene profiles, which include cytokines and other putative biomarkers that are indicative of infection status [22,23]. These investigations also revealed that differential gene expression patterns were identifiable irrespective of whether PBMC were stimulated with antigen. Differential gene expression patterns may therefore provide useful novel diagnostic and prognostic tools [20,24].

We have previously used a bovine targeted immunospecific cDNA microarray to study gene expression changes in PBMC from bovine tuberculosis-(BTB-) infected cattle cultured *in vitro *in the presence of bovine and avian tuberculins [25]. Stimulation with tuberculin antigens induced significant expression changes in a range of immune genes. In addition, the pattern of expression of many other genes provided evidence of an *M. bovis*-specific signature of infection. In the present study, we have used an expanded microarray platform to investigate gene expression differences that exist between infected and healthy control cattle *in vivo*, in the absence of *in vitro *antigenic stimulation. The results have yielded insights regarding the immune response to bovine tuberculosis, indicating that the expression of innate immune genes in *in vivo *infected animals is suppressed. This innate immune gene repression may limit the initiation of an appropriate adaptive immune response, which may contribute to progression of the disease. This study has demonstrated the involvement of a number of genes previously not associated with host defence or inflammation and has used stringent microarray analysis methods to detect and validate a robust gene signature of infection. The results highlight the usefulness of large-scale genomics approaches to detect biomarkers of disease and gene signatures of infection that in future may form the basis for novel diagnostics and/or therapeutics.

## Results

### Analysis of leukocyte cell population subsets and IFN-γ release from control and BTB-infected animals

**Figure 1:**
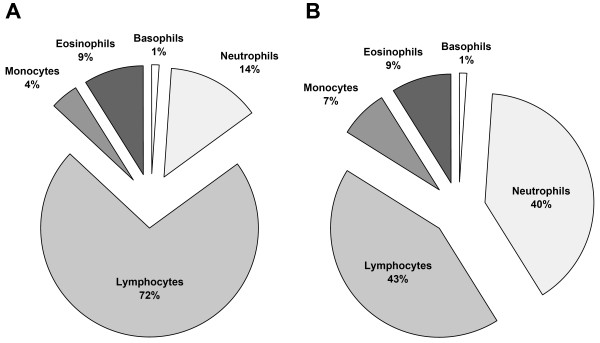
**Analysis of leukocyte cell population subset**. Analysis of leukocyte cell population subsets were performed on whole blood sampled *in vivo *for BTB-infected (A) and healthy control cattle (B). The lymphocyte and monocyte subpopulations are retained in peripheral blood mononuclear cells (PBMC).

The infected animals used in this study were chosen on the basis of their large responses to the comparative tuberculin skin test. The IFN-γ levels measured in whole blood of the infected animals were at least 25-fold greater than in the healthy control cattle (*P *< 0.001, data not shown), demonstrating that the infected animals were generating strong cell mediated immune responses. At post-mortem, each of the infected animals displayed gross tuberculosis lesions and were classified as being in the advanced stage of clinical disease. To rule out gene expression changes that might be attributable to differences in leukocyte populations between infected and control animals, whole blood samples were subjected to haematological analysis. There was no statistically significant difference in total white blood cell (WBC) counts between control and BTB-infected cattle (*P *= 0.721). However, neutrophil counts were significantly decreased and lymphocytes were significantly increased in BTB-infected cattle (*P *= 0.002 and *P *< 0.001 respectively, Fig. 1). Lymphocytes represented 72.4% of cells present in the total WBC samples from the BTB-infected group, but only 43.9% of WBC from the control group. A small reduction in the proportion of monocytes from 7% in the control animal samples to 4% in the BTB-infected animal samples was also observed.

### Microarray gene expression profile in BTB-infected cattle

Microarray analysis of mRNA was compared in the PBMC of six *M. bovis *infected cattle and six non-infected controls to investigate differential gene expression. The expression data generated from the microarray experiment were deposited in the NCBI Gene Expression Omnibus (GEO) repository [26] with experiment series accession GSE8857.

**Figure 2:**
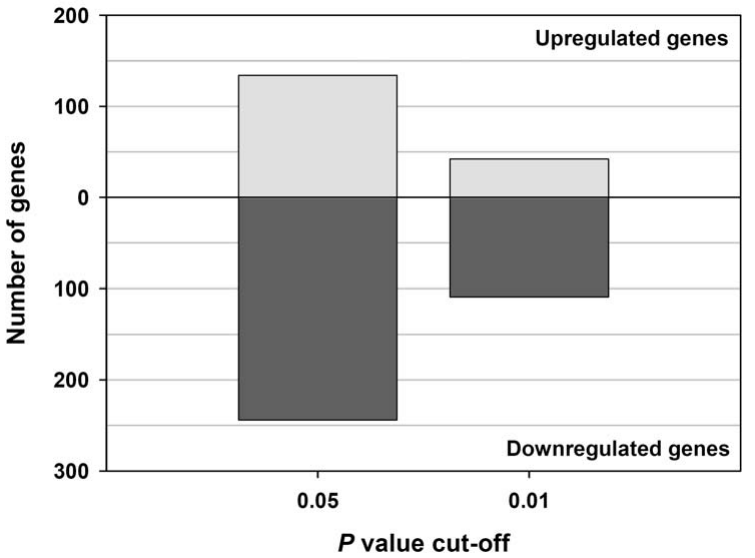
**Differentially expressed genes between BTB-infected and control cattle**. Statistically significant differentially expressed gene spot features between PBMC samples from BTB-infected (*n *= 6) and uninfected control animals (*n *= 6) *in vivo *at two different alpha levels (*P *≤ 0.05 and *P *≤ 0.01). For each *P *value, the number of genes with increased or decreased expression is shown for the BTB-infected animals relative to the control animals (see Additional files 1 and 2).

Of the 1,391 duplicated genes on the BOTL-5 microarray, 378 spot features showed significant differential expression between the BTB-infected and non-infected control animals at the *P *≤ 0.05 level (see Additional file 1). Of these, 151 were significant at *P *≤ 0.01 (Fig. 2) [see Additional file 1]. Among the 378 differentially expressed spot features, 134 were significantly increased in expression in BTB-infected animals (*P *≤ 0.05), and 244 spot features were significantly reduced in expression in BTB-infected animals (*P *≤ 0.05) compared to control animal samples. This trend was replicated at the *P *≤ 0.01 level (Fig. 2).

261 of the spot features represent 122 genes, where at least two replicate gene spot features were found to be significantly differentially expressed; 21 of these genes were identified as BOTL clones with no current gene match (see Additional file 2).

Furthermore, 90 of the 122 genes were expressed at lower levels in BTB-infected animals compared with non-infected controls. Among the genes reduced in expression with immune-related functions were *PRKCB1*, *PRKCA*, *AKT1*, *AKT2*, *EEF2*, *EEF1G*, *GATA4 *and *IER5*. Other genes normally associated with a proinflammatory immune response including *CSF2 *(-3.67 fold), *CD14 *(-3.08 fold), *CCL1 *(-4.86 fold), *CHUK *(-1.85 fold), *NFKB1 *(-2.89 fold), *TBK1 *(-1.63 fold), *MIF *(-1.91 fold), *CCR7 *(-2.49 fold), *BOLA *(-4.32 fold) and *BOLA-DRA *(-1.69) genes all displayed lower expression levels in BTB-infected animals relative to the control animal group (*P *≤ 0.05).

Messenger RNA (mRNA) transcripts for only 32 of the 122 genes showed higher levels of expression in BTB-infected animals. Most of these genes were EST sequences, the functions of which remain to be elucidated. Genes with increased expression and well characterised functions include the platelet-derived growth factor family, represented by the *PDGFA *and *PDGFB *genes (1.70 and 1.61 fold, respectively) and *ECGF1 *(1.77 fold). Also significantly increased were G protein-coupled receptor family 1 members *MCHR1 *(1.84 fold) and *GPR98 *(2.07 fold), a member of the receptor tyrosine kinase subfamily *AXL *(1.59 fold), a member of the Ig superfamily *CD84 *(1.53 fold) and the cytokine, *CCL15 *(1.60 fold) [and represented by replicate significant gene features at *P *≤ 0.05].

Fold change differences for differentially expressed genes on the microarray ranged from a decrease of 5.13 fold (the major histocompatibility complex, class I, A gene [*BOLA*] to an increase in expression of 2.14 fold (the growth arrest and DNA-damage-inducible, alpha gene [*GADD45A*]) in the BTB-infected cattle relative to control animals.

### Analysis of the microarray experimental false discovery rate (type 1 error)

Investigation of the experiment-specific false discovery rate (FDR) using exact multivariate permutations tests based on 462 available permutations demonstrated that the probability of obtaining at least 151 genes significant by chance (at the *P *≤ 0.01 level) if there are no real differences between the classes is 0.011. Furthermore, permutation-based analysis of the data using the Significance Analysis of Microarrays (SAM) package with 403 differentially expressed spot features (comparable to the 378 spot features obtained using conventional statistical analyses), demonstrated that only 15 of these 403 spot features were false positives (data not shown).

### Real time quantitative PCR (qRT-PCR) supports a trend of innate immune gene repression in BTB-infected cattle

**Figure 3:**
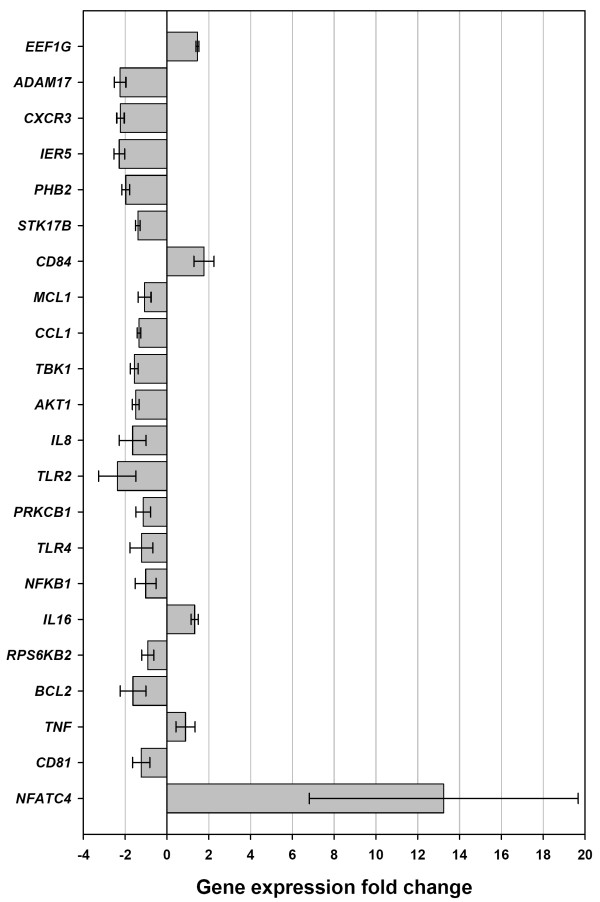
**Genes chosen for real time qRT-PCR data validation**. Shown are relative levels of differential gene expression confirmed between treatment groups *ex vivo *using real time qRT-PCR. Fold change values are shown for PBMC from BTB-infected cattle (*n *= 8) relative to PBMC from healthy control animals (*n *= 8). Error bars show the standard error of the mean for each gene.

An extended panel of 16 animals was used for real time qRT-PCR validation studies (BTB-infected cattle [*n *= 8] and control cattle [*n *= 8]). The 122 genes represented by significant replicate spot features were classified using gene ontology (GO). Selected genes from each GO class, supplemented with genes selected from relevant literature in human and murine models of TB were then used for these single gene expression studies are detailed in Table 1 (and shown in Fig. 3). Real time qRT-PCR data obtained for the following 17 genes; *EEF1G*, *CXCR3*, *IER5*, *PHB2*, *STK17B*, *CD84*, *MCL1*, *CCL1*, *TBK1*, *AKT1*, *PRKCB1*, *NFKB1*, *RPS6KB2*, *BCL2*, *TNF*, *CD81*, and *NFATC4 *corroborated the BOTL-5 microarray results obtained using RNA from the BTB-infected and control animals' PBMC (see Table 1 and Fig. 3). The most notable difference in gene expression was observed for the *NFATC4 *gene where its expression was increased by more than 13-fold in PBMC samples from BTB-infected cattle.

Expression levels of a number of genes involved in pathogen recognition, such as *TLR2 *and *TLR4*, as well as cytokine genes were also investigated by real time qRT-PCR (Fig. 3). The *TLR2 *and *TLR4 *genes were expressed at lower levels in BTB-infected animals compared with controls by -2.37 (*P *= 0.011) and -1.22 fold (*P *= 0.012) respectively. *ADAM17 *expression levels were also significantly lower (-2.24 fold, *P *= 0.001) in the BTB-infected animals. Gene expression levels for the *IL2*, *IL4 *and *IFNG *gene were not significantly different between BTB-infected and control cattle groups (*P *> 0.05), consistent with the microarray results. The interleukin 8 gene (*IL8*) was expressed at a significantly lower level in BTB-infected animal samples (-1.64 fold, *P *= 0.005). In contrast, the *IL16 *gene was significantly increased in BTB-infected animals (1.33 fold, *P *= 0.005) [see Table 1 and Fig. 3].

**Table 1:** Gene expression fold-change differences between BTB-infected animals (*n *= 8) and control animals (*n *= 8) using real time qRT-PCR

**Gene symbol**	**Gene name**	**Gene ontology (GO) function/s**	**Infected *vs *control group: relative expression**	***P*-value**
*EEF1G*	Eukaryotic translation elongation factor 1 gamma gene	Protein binding, translation elongation factor activity	1.46 ± 0.07	< 0.0001
*ADAM17*	ADAM metallopeptidase domain 17 (tumor necrosis factor, alpha, converting enzyme) gene	Metal ion binding, metalloendopeptidase activity, protein binding, zinc ion binding	-2.24 ± 0.28	< 0.0001
*CXCR3*	Chemokine (C-X-C motif) receptor 3 gene	C-X-C chemokine receptor activity, receptor activity, rhodopsin-like receptor activity	-2.22 ± 0.18	< 0.0001
*IER5*	Immediate early response 5 gene	Molecular function unknown	-2.28 ± 0.26	< 0.0001
*PHB2*	Prohibitin 2 gene	Estrogen receptor binding, protein binding, receptor activity, specific transcriptional repressor activity	-1.97 ± 0.19	< 0.0001
*STK17B*	Serine/threonine kinase 17b (apoptosis-inducing) gene	ATP binding, nucleotide binding, protein serine/threonine kinase activity, transferase activity	-1.39 ± 0.11	0.0007
*CD84*	CD84 antigen gene	Molecular function unknown	1.77 ± 0.48	0.0009
*MCL1*	Myeloid cell leukemia sequence 1 (BCL2-related) gene	Protein binding, protein channel activity, protein heterodimerization activity	-1.07 ± 0.31	0.0013
*CCL1*	Chemokine (C-C motif) ligand 1 gene	Chemokine activity	-1.33 ± 0.09	0.0014
*TBK1*	TANK-binding kinase 1 gene	ATP binding, nucleotide binding, protein serine/threonine kinase activity, signal transducer activity, transferase activity	-1.56 ± 0.19	0.0037
*AKT1*	V-akt murine thymoma viral oncogene homolog 1 gene	ATP binding, nucleotide binding, protein kinase activity, serine/threonine kinase activity, transferase activity	-1.49 ± 0.16	0.0042
*IL8*	Interleukin 8 gene	Chemokine activity, interleukin-8 receptor binding, protein binding	-1.64 ± 0.64	0.0048
*TLR2*	Toll-like receptor 2 gene	Gram-positive bacterial binding, lipopolysaccharide receptor activity, peptidoglycan binding, transferase activity	-2.37 ± 0.89	0.0108
*PRKCB1*	Protein kinase C, beta 1 gene	ATP binding, calcium ion binding, diacylglycerol binding, protein kinase C activity, transferase activity, zinc ion binding	-1.13 ± 0.35	0.0111
*TLR4*	Toll-like receptor 4	Lipopolysaccharide binding, protein binding, transferase activity, transmembrane receptor activity	-1.22 ± 0.55	0.0116
*NFKB1*	Nuclear factor of kappa light polypeptide gene enhancer in B-cells 1 (p105) gene	Protein binding, transcription factor activity	-1.02 ± 0.50	0.0228
*IL16*	Interleukin 16 (lymphocyte chemoattractant factor) gene	Cytokine activity, protein binding	1.33 ± 0.17	0.0326
*RPS6KB2*	Ribosomal protein S6 kinase, 70 kDa, polypeptide 2 gene	ATP binding, nucleotide binding, protein kinase activity, protein serine/threonine kinase activity, transferase activity	-0.92 ± 0.29	0.0342
*BCL2*	B-cell CLL/lymphoma 2 gene	Identical protein binding	-1.62 ± 0.61	0.0395
*TNF*	Tumor necrosis factor (TNF superfamily, member 2) gene	Protein binding, tumor necrosis factor receptor binding	0.89 ± 0.46	0.0426
*CD81*	CD81 molecule gene	Protein binding	-1.23 ± 0.41	0.0477
*NFATC4*	Nuclear factor of activated T-cells, cytoplasmic, calcineurin-dependent 4 gene	Transcription coactivator activity, transcription factor activity	13.22 ± 6.42	0.0482

Relative expression fold change values are shown with standard errors. Also shown are *P*-values from *t*-tests between the two groups.

### Cluster analysis identifies a gene expression signature of BTB infection

**Figure 4:**
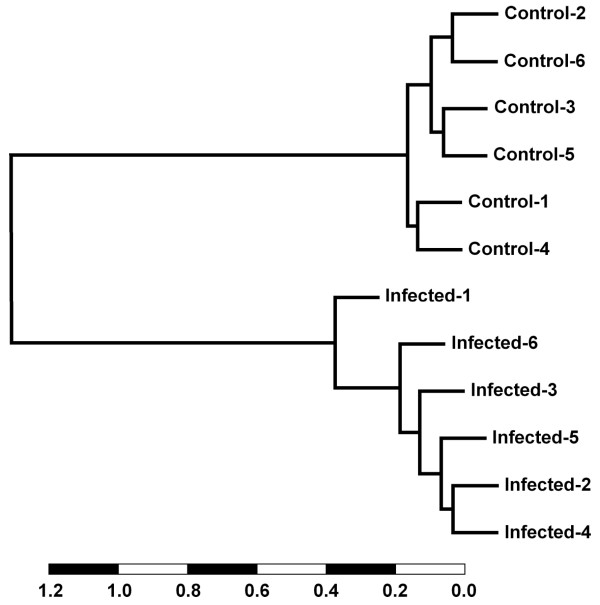
**A gene expression signature of BTB infection**. Hierarchical cluster dendrogram constructed with pairwise Pearson correlations from BOTL-5 microarray expression data. Data from 15 genes differentially expressed at the *P *≤ 0.001 level were used to construct the dendrogram (scale is expressed as units of the Pearson correlation).

A hierarchical cluster dendrogram was constructed for the 12 animals screened with the BOTL-5 microarrays using the expression data from a panel of the 15 most significant differentially expressed genes (*P *≤ 0.001). The results of this hierarchical clustering are presented in Fig. 4 and further details for the 15 genes used are provided in Table 2. This analysis of the expression of these 15 genes differentiated between both animal groups and resolved the disease status of the 12 animals.

**Table 2:** List of 15 genes significantly differentially expressed at the *P *< 0.001 level between BTB-infected cattle (*n *= 6) and control cattle (*n *= 6) from the BOTL-5 microarray data

**Array feature/Clone ID**	**Gene symbol**	**Gene name**	**Gene ontology (GO) function/s**	**Infected *vs *control animal relative expression**
NBFGC_AW656075	*NCOR1*	Nuclear receptor co-repressor 1	DNA binding, protein binding, transcription corepressor activity	-2.12
NBFGC_BF604459	*PPP2R5B*	PP2A protein phosphatase 2A B56-beta	Protein phosphatase type 2A regulator activity	1.43
BOTL0100001XG10R	*UCP2*	Uncoupling protein 2 (UCP2) (mitochondrial, proton carrier)	Binding, transporter activity	-1.80
BOTL0100002XD04R	*UNC84B*	Unc-84 homolog B	Microtubule binding	-1.58
BOTL0100003XB12R	*ZDHHC19*	Zinc finger, DHHC-type containing 19	Acyltransferase activity, metal ion binding, transferase activity, zinc ion binding	1.94
BOTL0100003XF01R	*NFKB1*	Nuclear factor of kappa light polypeptide gene enhancer in B-cells 1	Protein binding, transcription factor activity	-2.28
BOTL0100004XD01R	*GAN*	Giant axonal neuropathy (gigaxonin)	Protein binding	-1.41
BOTL0100005XF07R	*SFPQ*	Splicing factor proline/glutamine rich (polypyrimidine tract binding protein associated)	DNA, RNA, nucleotide and protein binding	-1.67
BOTL0100007_C06	*NRM*	Nurim	Nuclear envelope membrane protein	-3.07
BOTL0100013_F01	-	Unknown	Unknown – limited similarity to Formin 2	-1.59
Fibroblast growth factor receptor 1	*FGFR1*	Fibroblast growth factor receptor 1	ATP, nucleotide and protein binding. Receptor and tranferase activity.	-2.77
NBFGC_BE479784	*TBK1*	TANK-binding kinase 1	ATP and nucleotide binding. Protein kinase and signal transducer activity.	-1.63
NBFGC_BE682784	*28S*	28S ribosomal RNA gene	Protein biosynthesis	1.52
NBFGC_BF076990	GPR98	G protein-coupled receptor 98	G-protein coupled receptor activity, calcium ion binding	2.07
Neuropilin 1 (NRP1)	*NRP1*	Neuropilin 1	Receptor activity, vascular endothelial growth factor receptor activity	-2.98

Clone IDs were obtained from the Center for Animal Functional Genomics (CAFG) website [51].

The 15 genes used for the cluster analysis, expression for four of which was increased in the infected animals, included some genes with functions that are not well described in any species. These include the *NRM *(nurim [nuclear envelope membrane protein]), *ZDHHC19 *(zinc finger, DHHC-type containing 19), *UCP2 *(uncoupling protein 2 [mitochondrial, proton carrier]) and *GAN *(giant axonal neuropathy [gigaxonin]) genes. However, the panel also included well characterized genes of immunological relevance such as the *FGFR1 *(fibroblast growth factor receptor 1) gene, the transcription factor *NFKB1 *gene and the *TBK1 *gene, a mediator of the action of NF-κB.

Estimates of the experimental FDR using the SAM package and permutation analyses showed that the probability of getting at least 17 genes significant by chance at the *P *≤ 0.001 level, if there are no real differences between the classes, was 0.013 (data not shown). Furthermore, 13 of these 15 genes were represented by two or more significant gene features in the original BOTL-5 data. The real time qRT-PCR verification performed for the *NFKB1 *and the *TBK1 *genes, both of which are represented in the infection signature panel, supports the reliability of this method for the detection of a gene infection signature for BTB (see Table 2 and Fig. 3).

The accuracy of the 15 genes, estimated to be predictive of disease status in peripheral blood was further analyzed using leave-one-out cross-validation (LOOCV) [27] as implemented in BRB ArrayTools version 3.0. LOOCV analysis confirmed these gene predictors using a number of analyses including the diagonal linear discriminant, which classified the gene infection signature with a sensitivity and specificity of 0.833 between BTB-infected and control animal groups (data not shown). From the 15 gene list, 100% correct classification rate was obtained for the expression levels of four genes: *NCOR1*, *ZDHHC19*, *GAN *and an unknown gene represented by clone identifier BOTL0100013_F01 (Table 2).

## Discussion

The inability of infected cattle to eliminate *M. bovis *suggests that the host immune response is inadequate to control infection in these animals. The specific immune cell signalling pathways that are involved in the immune response to intracellular infectious agents are highly complex and poorly characterized in cattle. Although cell-mediated immunity is known to be critical for the control of mycobacterial infections; the role of the innate immune system has only recently been addressed in human and murine studies [11,8,9]. Cells and molecules of the innate immune system play a fundamental role in the detection of pathogen-associated molecular patterns, in phagocytosis, pathogen destruction, antigen presentation to T-lymphocytes that drive the production of proinflammatory cytokines, and the subsequent activation of an effective adaptive immune response. Interference in, or suppression of these molecular mechanisms, due to a change in the balance of cytokines, or in the pathogen-induced suppression of cell regulatory pathways may be a decisive factor in determining the progression of *M. bovis *infection in cattle [5,7,28].

The BTB-infected animals used in this study displayed a significant 29% increase in the relative proportion of lymphocytes in their blood (*P *< 0.001, Fig. 1), accompanied by a small decrease in the proportion of monocytes (4%). The production of IFN-γ after *in vitro *antigenic stimulation indicated the presence of *M. bovis*-specific T-lymphocytes in the BTB-infected lymphocyte populations. However, despite the influx of lymphocytes, the gene expression data presented here did not reveal a proinflammatory immune response in PBMC from these BTB-infected animals *in vivo*. In addition, the BOTL-5 microarray results showed that genes detected with decreased expression outnumbered genes detected with increased expression by a factor of two, suggesting gene repression (Fig. 2).

Estimation of the microarray platform-specific false discovery rate (FDR) provided information regarding the reliability of the 378 differentially expressed spot features detected using the BOTL-5 microarray platform under the specific experimental design and conditions. With 1,391 genes spotted on the array approximately 70 and 14 false positive genes would be expected using standard statistical tests at the *P *≤ 0.05 and *P *≤ 0.01 levels respectively. However, because each gene on the BOTL-5 microarray is represented twice, individual spot features are not strictly independent observations. Furthermore, the array is a targeted immunospecific platform, and as such, represents a subset of bovine genes that are known to participate in the immune response and ancillary processes. Therefore, there is likely to be a relatively high degree of functional overlap and co-regulation, such that many of these genes are not strictly independent of one another. Previous work has shown that the experimental FDR for a porcine brain microarray platform followed statistical expectations without the addition of a fold cut-off and that additional selection criteria could be used to virtually eliminate false positives [29]. Taken together, the analyses of the FDR in this study suggest that the microarray data is reliable and that the FDR was lower than random expectations with the experimental conditions described.

Ninety of the 122 genes represented by significant replicate spot features were expressed at lower levels in BTB-infected animals (Fig. 2). Furthermore, the 25 genes examined by real time qRT-PCR confirmed the BOTL-5 microarray results and supported an overall trend of repression of the immune response, which may be manifested primarily through decreased expression of innate immune genes (Fig. 3). Reduced expression of key indicator genes, with well established roles in the bovine immune response to BTB, associated with BTB-infection was particularly suggestive of innate immune gene repression *in vivo*. The expression of Toll-like receptor genes, *TLR2 *and *TLR4 *was reduced by 2.4-fold and 1.2-fold respectively in PBMC from the BTB-infected animals *ex vivo *(see Table 1 and Fig. 3). These results suggest that TLR expression associated with pathogen recognition and reaction to mycobacteria [12] was suppressed in PMBC of animals with advanced BTB infection. Significantly, the *NFKB1 *gene, a central mediator of the proinflammatory immune response and a gene that encodes a mediator of NF-κB action (*TBK1*) were both expressed at significantly reduced levels in BTB-infected animals with *P *values of 0.023 and 0.004, respectively (see Table 1 and Fig. 3). In addition, the microarray data indicated reduced expression of *CHUK*, a gene that also regulates NF-κB activation, (*P *= 0.005), further supporting the trend of immune gene repression in the BTB-infected animals. NF-κB is a key transcription factor for many of the genes involved in the immune response [30], and as such may be a key mediator of the gene repression detected in PBMC from the BTB-infected group.

The *CCL1 *gene, which encodes a cytokine that displays chemotactic activity for monocytes [31] also exhibited reduced expression (Fig. 3). Two genes that encode members of the G protein-coupled receptor family involved in chemotactic T-cell migration, dendritic cell maturation and recruitment of inflammatory cells (*CCR7 *and *CXCR3*) [32] are also expressed at significantly lower levels in BTB-infected animals based on the microarray data.

Expression of the *IL8 *gene, which encodes a neutrophil recruiting chemokine – a key mediator of the inflammatory response – was also reduced (Fig. 3). This observation was consistent with the reduced *NFKB1 *gene expression in the infected animals; NF-κB is a well characterised mediator of *IL8 *expression [33]. Furthermore, despite the relative expansion of lymphocytes in the PBMC from BTB-infected cattle (Fig. 1), a majority of genes are expressed at lower levels with no change in the expression of the proinflammatory *IFNG*, *IL2 *or *IL4 *genes detected using the BOTL-5 microarray or real time qRT-PCR (*P *= 0.487, 0.772 and *P *= 0.385 respectively for qRT-PCR results).

Recent studies of human tuberculosis infection demonstrate that mycobacteria can target cell-signalling pathways to regulate gene expression and subvert the host immune response [reviewed in reference [13]]. One particular study showed that mycobacteria specifically target the CD209 (DC-SIGN) molecule causing impaired dendritic cell maturation and induction of anti-inflammatory cytokines that promote immunosuppression [34]. In addition, other work has suggested that immune cell signalling suppression may be mediated through TLR-2 [15]. Both of these mechanisms could contribute to the survival of the mycobacteria.

Previous work using mycobacterial infections has demonstrated differential expression of TLR-2, TLR-4 [9,35], inflammatory cytokines including IFN-γ [36,37] and IL-8 [38-41], and BOLA MHC molecules [42,43]. In the present study, there was no discernible difference in expression for proinflammatory molecules between the BTB-infected and control animal groups. This observation suggests that PBMC from BTB-infected cattle display different gene expression program compared to both the healthy control animals and to PBMC exposed to *M. bovis *antigens *in vitro *[25].

The differences in cell subpopulations shown between the BTB-infected and control animal group (Fig. 1) may contribute to some of the gene expression changes detected; however, the data presented here also supports the hypothesis that a host- or pathogen-driven process of innate immune gene repression in BTB-infection *in vivo *is responsible for the progression of the disease. These results are consistent with recent work involving Johne's disease in cattle caused by *M. avium *subsp. *paratuberculosis *(MAP), where suppression of the immune response was detected in late stage infection animals [44] and a novel gene expression program was identified for PBMC *in vivo *[22].

One of the aims of this study was to extract gene expression patterns that are associated with host-pathogen interactions, and that can be interrogated to identify a robust pathogen-specific molecular signature of infection [20,45]. It is clear that this approach could be problematic because natural gene expression variation for individual animals and their response to *M. bovis *infection has not been characterized [6,28]. However, gene expression infection signatures do hold promise; a recent study showed that human gene expression differences due to disease state were significantly greater than variation due to natural factors such as age and gender [46]. In addition, Coussens and co-workers have established two genes (*TNFSF8 *and *SELP*) in a gene infection signature for Johne's disease in cattle [23].

The results presented here suggest that gene expression differences for key immune genes identified using the BOTL-5 microarray and verified using real time qRT-PCR play a role in disease pathogenesis and importantly, that these genes may serve as biomarkers for BTB-infection status. Cluster analysis identified a panel of 15 genes indicative of disease status in PBMC from naturally infected animals, in the absence of antigenic stimulation with tuberculin. In addition, results from class prediction analyses allocated a sensitivity and specificity score of 83% for these gene classifiers as predictive of disease status for the two groups of animals used. Taking these observations into consideration, these genes may therefore represent robust and stable biomarkers for BTB infection. We are currently investigating the sensitivity and specificity of this gene infection signature in a larger cohort of naturally infected and uninfected cattle.

## Conclusion

The results from the present study support a primary trend of innate immune gene repression in PBMC from BTB-infected animals. Additionally, a distinct gene expression profile that is predictive of disease state is evident, that also sheds light on the cell regulatory pathways associated with pathogenesis of bovine tuberculosis. However, it is important to note that different patterns of gene expression may be evident in tissues at the sites of active infection. Also, some of the gene expression changes we observed may not be specific for *M. bovis *infection and may represent a general phenomenon associated with other advanced stage infections or pathologies.

This study highlights the importance of the natural host for *M. bovis *infection as a model to investigate the immune response to tuberculosis using functional genomics technologies. Genes and cellular regulatory pathways involved in the bovine innate immune response to tuberculosis will likely show evolutionary overlap with mechanisms of response to *M. tuberculosis *in humans. These results also suggest that clinical strategies that target novel innate immune molecules might be useful in combating mycobacterial infections by shifting the balance between immune activation and suppression to favour the elimination of pathogens.

## Methods

### Experimental animals

Sixteen cattle were used for this study. The eight infected animals were chosen from herds with a recent history of chronic infection with *M. bovis*. The animals were selected on the basis of the skin-fold thickness response to bovine and avian tuberculin in the single intradermal comparative tuberculin test (SICTT). The SICTT reactor animals were selected where the skin-fold thickness response to PPD-bovine exceeded that of PPD-avian by at least 12 mm. All of these animals were also measured positive in a whole blood IFN-γ assay [47]. The cattle were confirmed positive for tuberculosis following detailed *post-mortem *pathological examination and/or culture. Bronchial, mediastinal, submandibular, retropharyngeal, mesenteric and hepatic lymph nodes and lungs were examined macroscopically for tuberculosis lesions. Suspected lesions were cultured on Stonebrinks and Lowenstein-Jensen media at 37°C for eight weeks to detect *M. bovis *[48]. The eight non-infected control animals were selected from a herd without a recent history of tuberculosis and were SICTT and IFN-γ test negative.

### Blood sampling and analysis

400 ml of blood was collected from each animal in sterile heparinised bottles. Five ml of blood was used for haematological analysis using an Abbott CELL-DYN 3500R automated haematology analyzer (Abbott Laboratories). Leukocyte cell population subsets were compared between infected and control groups (*n *= 8) using Student's *t*-test.

### PBMC separation, RNA extraction and quality control

PBMC were isolated using the Percoll™ gradient method with a standard protocol [49]. PBMC were seeded at 10^7 ^per culture plate and cultured in RPMI 1640 medium supplemented with 5% FBS, 0.1% mercaptoethanol and 0.1% gentamicin. All PBMC samples were cultured overnight at 37°C in 5% CO_2_. Overnight culture was carried out to minimise noise in gene expression measurements potentially introduced by the mechanical disruption of cells associated with PBMC isolation. Residual cells not seeded for culture were immediately suspended in 3 ml TriReagent^® ^(Molecular Research Centre Inc.) and frozen in 1.5 ml cryotubes at -80°C for use later as a common reference RNA (CRR) pool. Total RNA was extracted using a combined TriReagent^®^, DNase treatment and Qiagen RNeasy^® ^method (Qiagen Ltd.) according to the manufacturers' instructions. The integrity and stability of RNA samples is crucial for gene expression analyses using microarray technology; therefore, RNA yield and quality were assessed using an Agilent 2100 Bioanalyzer (Agilent Technologies). The two-step method for RNA extraction described above was found to produce RNA of high yield and quality (ratios of 18S to 28S ribosomal RNA averaged > 1.6).

### Microarray experimental design

The 3,888 feature BOTL-5 immunogenetic cDNA microarray system used has been described previously [50]. Technical information with gene content and sequence information for the BOTL-5 array can be downloaded from the 'Links' section on the MSU Center for Animal Functional Genomics website [51]. The NCBI GEO platform accession for the BOTL-5 microarray is: GPL5751. The immunobiology-targeted BOTL-5 array contains 1,391 genes or ESTs spotted in duplicate with multiple additional control features (blank spots, negative spots, housekeeping genes) and is an expanded version of the BOTL-4 array described previously [52,25]. A reference design was used for microarray hybridizations, such that all RNA samples were labelled using Cy3 and co-hybridized with Cy5 labelled CRR pool. It was hypothesized that the CRR pool would display similar mRNA expression levels and gene coverage as the target samples, therefore allowing accurate and consistent comparison of gene expression data without arbitrarily pairing animals from the two groups [53]. The CRR pool contained equal amounts of total RNA from the treated and control animal groups. Twelve arrays were hybridized in total, representing six individual animals from each treatment group.

### cDNA labelling, hybridisation and scanning

cDNA synthesis, Cy3 and Cy5 labelling and microarray hybridizations were performed as previously described [25] with the following modifications. Each labelling reaction contained a total of 8 μg total RNA per sample and 10 μg total RNA from the CRR. Labelled cDNAs were purified to remove unincorporated dyes using a QIAquick^® ^purification kit (Qiagen Ltd.) and concentrated using Microcon^® ^centrifugal filter devices (Millipore Ltd.) according to the manufacturers' instructions. Labelled samples were combined (either an infected or a control sample combined with a CRR sample) and co-hybridized on the BOTL-5 microarrays using SlideHyb Glass Array Hybridization Buffer #3 (Ambion Ltd.). Microarray hybridizations were performed using a Tecan HS400 hybridisation station (Tecan Ltd.) with the following protocol – Step 1: 75°C, wash 10 s, soak 20 s, 1 cycle; probe injection: 85°C; denaturation: 95°C, 2 min; hybridization cycle 1: 65°C, time 35 min, agitation frequency medium; hybridization cycle 2: 55°C, time 35 min, agitation frequency medium; hybridization cycle 3: 50°C, time 2 h 30 m, agitation frequency medium; wash cycle 1: 42°C, wash 10 s, soak 20 s, 2 cycles; wash cycle 2: 33°C, wash 15 s, soak 30 s, 2 cycles; wash cycle 3: 33°C, wash 20 s, soak 40 s, 2 cycles; slide drying: 30°C, 1 min 30 s. Microarrays were scanned immediately using a GenePix 4000B microarray scanner (Molecular Devices Ltd.). Data was captured using GenePix Pro version 5.0 software (Molecular Devices Ltd.).

### Data processing, normalization and analysis and clustering

The working signal intensities were generated using the mean foreground intensity values minus the median background intensity values as outputted from the GenePix Pro 5.0 results file. Two methods of data pre-processing were used to flag unreliable data. If the signal intensity in one channel was less than 100 and if the signal intensity for the other channel is less than 200, the spot was flagged. If the signal intensity in one channel was less than 100 and the signal intensity was larger than 200 in the second channel, 100 was assigned to the intensity of the first channel.

Median-based normalization corrects the data such that all arrays have the same median [54]. The median value is less likely to be influenced by outlying values. A normalization factor was calculated by summing the intensities in both channels and adjusts both ratios to ensure a median of 1.0. Therefore, the median log expression ratio for all features on the array was adjusted to zero (corresponding to an expression ratio of 1.0). The formula used for median normalization is as follows:

Cjk≡Cj=mediani∈S(log⁡(RjkGjk))
 MathType@MTEF@5@5@+=feaafiart1ev1aaatCvAUfKttLearuWrP9MDH5MBPbIqV92AaeXatLxBI9gBaebbnrfifHhDYfgasaacPC6xNi=xI8qiVKYPFjYdHaVhbbf9v8qqaqFr0xc9vqFj0dXdbba91qpepeI8k8fiI+fsY=rqGqVepae9pg0db9vqaiVgFr0xfr=xfr=xc9adbaqaaeGacaGaaiaabeqaaeqabiWaaaGcbaGaem4qam0aaSbaaSqaaiabdQgaQjabdUgaRbqabaGccqGHHjIUcqWGdbWqdaWgaaWcbaGaemOAaOgabeaakiabg2da9maaxababaGaemyBa0MaemyzauMaemizaqMaemyAaKMaemyyaeMaemOBa4galeaacqWGPbqAcqGHiiIZcqWGtbWuaeqaaOWaaeWaaeaacyGGSbaBcqGGVbWBcqGGNbWzdaqadaqaamaalaaabaGaemOuai1aaSbaaSqaaiabdQgaQjabdUgaRbqabaaakeaacqWGhbWrdaWgaaWcbaGaemOAaOMaem4AaSgabeaaaaaakiaawIcacaGLPaaaaiaawIcacaGLPaaaaaa@515E@

Where *C*_*jk *_represents the normalisation factor, *S* the set of genes for normalization, *R*_*jk*_the observed log ratio of the red (Cy5) channel and *G*_*jk*_the observed log ratio of the green (Cy3) channel [55,56].

Microarray data analysis was carried out using class comparisons between experimental groups (parametric *t*-tests) as implemented in BRB ArrayTools version 3.0 [57].

### Microarray platform-specific false discovery rate

The false discovery rate (FDR) for the microarray data was investigated using permutation analysis. Data sets for individual samples were randomly assigned control or infected status to produce new permuted data. This procedure was carried out for 1,000 permutations and the number of differentially expressed genes for each permuted data set was then estimated using BRB ArrayTools. In addition, the experiment-specific FDR was further examined using the Significance Analysis of Microarrays (SAM) version 2.0 package [58].

### Supervised cluster analysis

Gene expression profiles for each animal were clustered using average linkage hierarchical clustering implemented in the BRB ArrayTools version 3.0 package with pairwise Pearson correlations as the distance metric. To test the accuracy of the clustered class predictors whose expression state changed between classes at the *P *≤ 0.001 level, a method of class prediction was used as implemented in BRB ArrayTools. Leave-one-out cross-validation (LOOCV) was performed to test the accuracy of each class predictor and compared to the probability of a correct class prediction by chance alone, based on the *P*-value and total number of genes analyzed [27].

### Real time quantitative reverse transcription PCR

Replicate spot features on the BOTL array were used as a check for the quality control of gene expression data. Each spot was analyzed individually thereby allowing the individual genes to be flagged if expression results from two or more replicates were statistically different. This enabled the identification of differentially expressed genes that had a low probability of being false positives and expedited the choice of target genes for real time qRT-PCR validation of the microarray results. The H3 histone family 3A (*H3F3A*) gene was used as a quantitative reverse transcription PCR (qRT-PCR) reference gene for the present study. This gene displayed the least gene expression differences among the 12 control and BTB-infected samples analyzed using the BOTL microarray platform (data not shown).

Gene expression differences detected using the BOTL microarray platform were validated using a MX3000P™ fluorescence detection real-time PCR system (Stratagene Europe). Total RNA samples from each of the 16 samples (representing 8 animals per treatment group) were converted into first strand cDNAs with the following protocol: 2 μg of each RNA was combined with 10 mM oligo (dT)_12–18 _primer and DNase/RNase-free sterile water in a 10-μl volume that was incubated for 5 min at 70°C followed by 5 min at 20°C. A mastermix containing 200 U M-MuLV reverse transcriptase (New England Biolabs Ltd.), 2 μl of reaction buffer (final reagent concentrations of 50 mM Tris-HCl, pH 8.3, 75 mM KCl, 3 mM MgCl_2 _and 10 mM DTT), and a final concentration of 0.5 mM each dNTP were added to obtain a final reaction volume of 20 μl. Reverse transcription was allowed to proceed at 42°C for 60 min and then samples were heated to 72°C for 15 min, cooled to 37°C prior to the addition of 2 U of DNase-free RNAase H (Invitrogen Ltd.). Incubation at 37°C was continued for 20 min with RNase H to remove the original RNA template followed by enzyme inactivation with 0.5 μl of 0.5 M EDTA (pH 8.0). First-strand cDNAs were purified with QuickClean resin according to the manufacturer's instructions (BD Biosciences) followed by precipitation in 80% ethanol supplemented with 100 mM sodium acetate. Purified cDNAs were suspended in DNase/RNase-free sterile water, quantified using an Agilent Bioanalyzer (Agilent Technologies) and diluted to a final concentration of 10 ng/μl and stored at -80°C until required. Gene-specific oligonucleotide primer pairs were designed using Primer Express^® ^version 2.0 software (Applied Biosystems) and synthesized commercially (Invitrogen Ltd.). Experimental details for these primer pairs are shown in Table 3. Real-time qRT-PCR reactions were performed in 25 μl reaction volumes with 0.5 μl HOT FIREPol ^® ^DNA polymerase and buffer (Solis Biodyne Inc.), 2.5 μl of 10 × manufacturer's reaction buffer B, 2.5 μl of 25 mM MgCl_2 _and 2.5 μl of 2 mM dNTP mix, optimized primer mix and water made to 14.1 μl (final concentrations ranging from 100 – 900 nM each, Table 3), 1.25 μl of a 1/60,000 dilution of SYBR ^® ^Green I dye (BioGene Ltd.) and 2 μl (20 ng) of cDNA template. Real-time qRT-PCR amplification conditions were always 95°C for 1 min followed by 40 cycles of 95°C for 15 s then 60°C for 60 s. All reactions were performed in duplicate and amplicons for the *H3F3A *reference gene mRNA transcript were used to normalize expression data for the target genes.

**Table 3:** Real time qRT-PCR primer sequences, optimum primer concentrations and amplicon sizes for all validated genes.

**Array feature/Clone ID**	**Gene symbol**	**Forward primer (5'-3')**	**Reverse primer (5'-3')**	**Amplicon size (bp)**	**Primer conc. (nM)**
Reference gene	*H3F3A*	CATGGCTCGTACAAAGCAGA	ACCAGGCCTGTAACGATGAG	136	100
PCR Amplicon	*ACTB*	AAGCCGGCCTTGCACAT	TAACTCGAGAGCCAACGTCTCC	66	900
NBFGC_BE752490	*ADAM17*	TCAAAGTCGTGGTGGTAGATGG	AATTAGTCTCCAAAGCGGCTCT	188	900
NBFGC_AW656779	*AKT1*	GAGTACTTCAGGGCCGTCAG	GGTGATCCTGGTGAAGGAGA	160	900
PCR Amplicon	*BCL2*	ATGACTTCTCTCGGCGCTAC	ATGACCGAGTACCTGAACCG	244	300
PCR Amplicon	*CCL1*	AGGCTGGATCTGCTCCCAAAT	GGTGATGTGTGCAAGTTCACCA	152	900
BOTL0100003XA07R	*CD81*	TTCATGTCCTGAAGCTCCCTGT	TGAAGGCATAAGGCTGCTCGT	284	300
BOTL0100013_C12	*CD84*	TAAGTGGTGTGTCATGGCAGGT	GGCTGGAGGCTGAATATGACTG	103	300
PCR Amplicon	*CXCR3*	GAAAGCAGTGTGGACATAGCCA	CGGAACTTGACACCCACAAAG	101	900
NBFGC_BF230159	*EEF1G*	TGGATGCTCACTTGAAGACG	ACTGGGCCATTTTCTCACAG	222	300
PCR Amplicon	*GAPDH*	CTCCCAACGTGTCTGTTGTG	TGAGCTTGACAAAGTGGTCG	222	300
BOTL0100013_E07	*IER5*	AAGACCCCCGAGACTTCG	ACACTCTTCAAGGCGGAGAG	115	300
PCR Amplicon	*IFNG*	TGATGGCATGTCAGACAGCA	GGCACAAGTCATATAGCCTGACAC	51	300
PCR Amplicon	*IL2*	CTTGCACTCGTTGCAAACG	CAAGCTCTCCAGGATGCATACA	183	300
PCR Amplicon	*IL4*	GCCACACGTGCTTGAACAAA	TCTCAACAGCTTGGCAAGCA	63	300
PCR amplicon	*IL8*	AGGTGGTGTTTGAAGCCCAT	CACAACCTTCTGCACCCACTT	123	900
PCR Amplicon	*IL16*	CGCGGTTTGAAGAATGGAAC	TCACAGGTCCATCAGGCAAC	51	300
PCR Amplicon	*MCL1*	AGGTGACTGAAAGGCCTGTCTC	CAACATGTGCCTCTTCTCCCT	244	900
BF775342 NFATC4	*NFATC4*	AACCACTGCCCCTCTCTGAAAC	CCTCGACCCCAGATCACAAAGA	107	300
BOTL0100003XF01R	*NFKB1*	ATACTGAACAATGCCTTCCGG	CACGTCAATGGCCTCAGTGTAG	135	300
BOTL0100013_G05	*PHB2*	GGCGGCGCGGATGT	AGGTTATATCAAGCTACGCAAGATCC	65	900
NBFGC_AW335987	*PRKCB1*	ATCGAGAGGGAGGTCCTCAT	GGTCTTGGTCTTCTGCTTGC	141	300
PCR Amplicon	*RPL19*	AATGCCCGAGAAGGTAACCTG	GGATATGTTCCATGAGGATCCG	164	100
NBFGC_AW669767	*RPS6KB2*	TGTGGAACTGGCCTATGCCTTC	AAGATGCCTTCTCGCTCCAGGT	105	300
PCR Amplicon	*STK17B*	ACAGGCCCTCTTGTAATGGCAC	AGCAAATCGGACACAAGCTCG	136	300
NBFGC_BE479784	*TBK1*	TGGACCAATTGACTGGAGTGGA	TGATCTGCCTCAAGGATGTTTG	105	300
Not represented	*TLR2*	CCATTGACAAGAAGGCCAT	AACCCTTCCTGCTGAGTCTCAT	107	900
Not represented	*TLR4*	CGAGAGCACCTATGATGCCTTT	ATGGCCACCCCAGGAATAAA	144	900
PCR Amplicon	*TNF*	TCTACCAGGGAGGAGTCTTCCA	GTCCGGCAGGTTGATCTCA	68	300

Clone IDs were obtained from the Center for Animal Functional Genomics (CAFG) website [51].

Real time qRT-PCR data were analysed using the 2^-ΔΔCt ^method [59] as described previously [25]. Real time qRT-PCR gene expression log_2 _values from both groups were compared using Student's *t*-test.

## Authors' contributions

KM was primarily responsible for experimental design, coordination, performance and validation of results. EG and EC provided access to animal samples and valuable expertise in analysis of results. MD and TF provided valuable assistance in processing of samples and provision of reagents. JK and COF provided important comments and discussion as well as manuscript editing. YZ carried out the microarray and LOOCV analyses. DM was responsible for experimental design, data analysis, and manuscript preparation and editing. All authors read and approved the final manuscript.

## Supplementary Material

Additional file 1 BOTL-5 microarray spot features that showed significant differential expression between the BTB-infected and non-infected control animals. 378 BOTL-5 microarray spot features that showed significant differential expression between the BTB-infected and non-infected control animals at the *P *≤ 0.05 level. Click here for file

Additional file 2 Genes on the BOTL-5 microarray represented by two or more replicate features that showed significant differential expression between the BTB-infected and non-infected control animals. 122 genes on the BOTL-5 microarray represented by two or more replicate features that showed significant differential expression between the BTB-infected and non-infected control animals at the *P *≤ 0.05 level. Click here for file

## References

[B1] Rothel JS, Jones SL, Corner LA, Cox JC, Wood PR (1992). The gamma-interferon assay for diagnosis of bovine tuberculosis in cattle: conditions affecting the production of gamma-interferon in whole blood culture. Aust Vet J.

[B2] Pollock JM, Buddle BM, Andersen P (2001). Towards more accurate diagnosis of bovine tuberculosis using defined antigens. Tuberculosis (Edinb).

[B3] Neill SD, Cassidy J, Hanna J, Mackie DP, Pollock JM, Clements A, Walton E, Bryson DG (1994). Detection of Mycobacterium bovis infection in skin test-negative cattle with an assay for bovine interferon-gamma. Vet Rec.

[B4] Gormley E, Doyle MB, McGill K, Costello E, Good M, Collins JD (2004). The effect of the tuberculin test and the consequences of a delay in blood culture on the sensitivity of a gamma-interferon assay for the detection of Mycobacterium bovis infection in cattle. Vet Immunol Immunopathol.

[B5] Pollock JM, McNair J, Welsh MD, Girvin RM, Kennedy HE, Mackie DP, Neill SD (2001). Immune responses in bovine tuberculosis. Tuberculosis (Edinb).

[B6] Pollock JM, Neill SD (2002). Mycobacterium bovis infection and tuberculosis in cattle. Vet J.

[B7] Pollock JM, Welsh MD, McNair J (2005). Immune responses in bovine tuberculosis: towards new strategies for the diagnosis and control of disease. Vet Immunol Immunopathol.

[B8] Reiling N, Holscher C, Fehrenbach A, Kroger S, Kirschning CJ, Goyert S, Ehlers S (2002). Cutting edge: Toll-like receptor (TLR)2- and TLR4-mediated pathogen recognition in resistance to airborne infection with Mycobacterium tuberculosis. J Immunol.

[B9] Heldwein KA, Liang MD, Andresen TK, Thomas KE, Marty AM, Cuesta N, Vogel SN, Fenton MJ (2003). TLR2 and TLR4 serve distinct roles in the host immune response against Mycobacterium bovis BCG. J Leukoc Biol.

[B10] Elass E, Aubry L, Masson M, Denys A, Guerardel Y, Maes E, Legrand D, Mazurier J, Kremer L (2005). Mycobacterial lipomannan induces matrix metalloproteinase-9 expression in human macrophagic cells through a toll-like receptor 1 (TLR1)/TLR2- and CD14-dependent mechanism. Infect Immun.

[B11] Heldwein KA, Fenton MJ (2002). The role of Toll-like receptors in immunity against mycobacterial infection. Microbes Infect.

[B12] Quesniaux V, Fremond C, Jacobs M, Parida S, Nicolle D, Yeremeev V, Bihl F, Erard F, Botha T, Drennan M, Soler MN, Le Bert M, Schnyder B, Ryffel B (2004). Toll-like receptor pathways in the immune responses to mycobacteria. Microbes Infect.

[B13] Koul A, Herget T, Klebl B, Ullrich A (2004). Interplay between mycobacteria and host signalling pathways. Nat Rev Microbiol.

[B14] Hestvik AL, Hmama Z, Av-Gay Y (2005). Mycobacterial manipulation of the host cell. FEMS Microbiol Rev.

[B15] Netea MG, Van der Meer JW, Kullberg BJ (2004). Toll-like receptors as an escape mechanism from the host defense. Trends Microbiol.

[B16] Russell DG, Sturgill-Koszycki S, Vanheyningen T, Collins H, Schaible UE (1997). Why intracellular parasitism need not be a degrading experience for Mycobacterium. Philos Trans R Soc Lond B Biol Sci.

[B17] Yao J, Burton JL, Saama P, Sipkovsky S, Coussens PM (2001). Generation of EST and cDNA microarray resources for the study of bovine immunobiology. Acta Vet Scand.

[B18] Xu Y, Xie J, Li Y, Yue J, Chen J, Chunyu L, Wang H (2003). Using a cDNA microarray to study cellular gene expression altered by Mycobacterium tuberculosis. Chin Med J (Engl).

[B19] Staudt LM, Brown PO (2000). Genomic views of the immune system. Annu Rev Immunol.

[B20] Campbell CJ, Ghazal P (2004). Molecular signatures for diagnosis of infection: application of microarray technology. J Appl Microbiol.

[B21] Blumenthal A, Lauber J, Hoffmann R, Ernst M, Keller C, Buer J, Ehlers S, Reiling N (2005). Common and unique gene expression signatures of human macrophages in response to four strains of Mycobacterium avium that differ in their growth and persistence characteristics. Infect Immun.

[B22] Coussens PM, Colvin CJ, Rosa GJ, Perez Laspiur J, Elftman MD (2003). Evidence for a novel gene expression program in peripheral blood mononuclear cells from Mycobacterium avium subsp. paratuberculosis-infected cattle. Infect Immun.

[B23] Skovgaard K, Grell SN, Heegaard PM, Jungersen G, Pudrith CB, Coussens PM (2006). Differential expression of genes encoding CD30L and P-selectin in cattle with Johne's disease: Progress toward a diagnostic gene expression signature. Vet Immunol Immunopathol.

[B24] Jenner RG, Young RA (2005). Insights into host responses against pathogens from transcriptional profiling. Nat Rev Microbiol.

[B25] Meade KG, Gormley E, Park SD, Fitzsimons T, Rosa GJ, Costello E, Keane J, Coussens PM, MacHugh DE (2006). Gene expression profiling of peripheral blood mononuclear cells (PBMC) from Mycobacterium bovis infected cattle after in vitro antigenic stimulation with purified protein derivative of tuberculin (PPD). Vet Immunol Immunopathol.

[B26] Barrett T, Troup DB, Wilhite SE, Ledoux P, Rudnev D, Evangelista C, Kim IF, Soboleva A, Tomashevsky M, Edgar R (2007). NCBI GEO: mining tens of millions of expression profiles--database and tools update. Nucleic acids research.

[B27] Bair E, Tibshirani R (2004). Semi-supervised methods to predict patient survival from gene expression data. PLoS biology.

[B28] Welsh MD, Cunningham RT, Corbett DM, Girvin RM, McNair J, Skuce RA, Bryson DG, Pollock JM (2005). Influence of pathological progression on the balance between cellular and humoral immune responses in bovine tuberculosis. Immunology.

[B29] Nobis W, Ren X, Suchyta SP, Suchyta TR, Zanella AJ, Coussens PM (2003). Development of a porcine brain cDNA library, EST database, and microarray resource. Physiol Genomics.

[B30] Ghosh S, May MJ, Kopp EB (1998). NF-kappa B and Rel proteins: evolutionarily conserved mediators of immune responses. Annu Rev Immunol.

[B31] Miller MD, Krangel MS (1992). The human cytokine I-309 is a monocyte chemoattractant. Proc Natl Acad Sci U S A.

[B32] Agace WW, Roberts AI, Wu L, Greineder C, Ebert EC, Parker CM (2000). Human intestinal lamina propria and intraepithelial lymphocytes express receptors specific for chemokines induced by inflammation. Eur J Immunol.

[B33] Hoffmann E, Dittrich-Breiholz O, Holtmann H, Kracht M (2002). Multiple control of interleukin-8 gene expression. J Leukoc Biol.

[B34] Geijtenbeek TB, Van Vliet SJ, Koppel EA, Sanchez-Hernandez M, Vandenbroucke-Grauls CM, Appelmelk B, Van Kooyk Y (2003). Mycobacteria target DC-SIGN to suppress dendritic cell function. J Exp Med.

[B35] Doherty TM, Arditi M (2004). TB, or not TB: that is the question - does TLR signaling hold the answer?. J Clin Invest.

[B36] Cooper AM, Dalton DK, Stewart TA, Griffin JP, Russell DG, Orme IM (1993). Disseminated tuberculosis in interferon gamma gene-disrupted mice. J Exp Med.

[B37] Khalifeh MS, Stabel JR (2004). Effects of gamma interferon, interleukin-10, and transforming growth factor beta on the survival of Mycobacterium avium subsp. paratuberculosis in monocyte-derived macrophages from naturally infected cattle. Infect Immun.

[B38] Zhang Y, Broser M, Cohen H, Bodkin M, Law K, Reibman J, Rom WN (1995). Enhanced interleukin-8 release and gene expression in macrophages after exposure to Mycobacterium tuberculosis and its components. J Clin Invest.

[B39] Wickremasinghe MI, Thomas LH, Friedland JS (1999). Pulmonary epithelial cells are a source of IL-8 in the response to Mycobacterium tuberculosis: essential role of IL-1 from infected monocytes in a NF-kappa B-dependent network. J Immunol.

[B40] Fietta A, Meloni F, Francioli C, Morosini M, Bulgheroni A, Casali L, Gialdroni Grassi G (2001). Virulence of Mycobacterium tuberculosis affects interleukin-8, monocyte chemoattractant protein-1 and interleukin-10 production by human mononuclear phagocytes. Int J Tissue React.

[B41] Song CH, Lee JS, Kim HJ, Park JK, Paik TH, Jo EK (2003). Interleukin-8 is differentially expressed by human-derived monocytic cell line U937 infected with Mycobacterium tuberculosis H37Rv and Mycobacterium marinum. Infect Immun.

[B42] Noss EH, Harding CV, Boom WH (2000). Mycobacterium tuberculosis inhibits MHC class II antigen processing in murine bone marrow macrophages. Cell Immunol.

[B43] Noss EH, Pai RK, Sellati TJ, Radolf JD, Belisle J, Golenbock DT, Boom WH, Harding CV (2001). Toll-like receptor 2-dependent inhibition of macrophage class II MHC expression and antigen processing by 19-kDa lipoprotein of Mycobacterium tuberculosis. J Immunol.

[B44] Coussens PM, Colvin CJ, Wiersma K, Abouzied A, Sipkovsky S (2002). Gene expression profiling of peripheral blood mononuclear cells from cattle infected with Mycobacterium paratuberculosis. Infect Immun.

[B45] Liu M, Popper SJ, Rubins KH, Relman DA (2006). Early days: genomics and human responses to infection. Curr Opin Microbiol.

[B46] Whitney AR, Diehn M, Popper SJ, Alizadeh AA, Boldrick JC, Relman DA, Brown PO (2003). Individuality and variation in gene expression patterns in human blood. Proc Natl Acad Sci U S A.

[B47] Rothel JS, Jones SL, Corner LA, Cox JC, Wood PR (1990). A sandwich enzyme immunoassay for bovine interferon-gamma and its use for the detection of tuberculosis in cattle. Aust Vet J.

[B48] Costello E, Quigley F, Flynn O, Gogarty A, McGuirk J, Murphy A, Dolan L (1998). Laboratory examination of suspect tuberculous lesions detected on abattoir postmortem examination of cattle from non-reactor herds. Irish Vet J.

[B49] Ulmer AJ, Scholz W, Ernst M, Brandt E, Flad HD (1984). Isolation and subfractionation of human peripheral blood mononuclear cells (PBMC) by density gradient centrifugation on Percoll. Immunobiology.

[B50] Coussens PM, Nobis W (2002). Bioinformatics and high throughput approach to create genomic resources for the study of bovine immunobiology. Vet Immunol Immunopathol.

[B51] MSU Center for Animal Functional Genomics website. http://www.nbfgc.msu.edu.

[B52] Evans AC, Ireland JL, Winn ME, Lonergan P, Smith GW, Coussens PM, Ireland JJ (2004). Identification of genes involved in apoptosis and dominant follicle development during follicular waves in cattle. Biol Reprod.

[B53] Novoradovskaya N, Whitfield ML, Basehore LS, Novoradovsky A, Pesich R, Usary J, Karaca M, Wong WK, Aprelikova O, Fero M, Perou CM, Botstein D, Braman J (2004). Universal Reference RNA as a standard for microarray experiments. BMC Genomics.

[B54] Quackenbush J (2002). Microarray data normalization and transformation. Nat Genet.

[B55] Simon RM, Korn EL, McShane LM, Radmacher MD, Wright GW, Zhao Y (2003). Design and analysis of DNA microarray investigations. Statistics for biology and health.

[B56] Zhao Y, Li MC, Simon R (2005). An adaptive method for cDNA microarray normalization. BMC Bioinformatics.

[B57] Simon R, Lam A, Li MC, Ngan M, Menenzes S, Zhao Y (2007). Analysis of gene expression data using BRB-Array Tools. Cancer Inform.

[B58] Tusher VG, Tibshirani R, Chu G (2001). Significance analysis of microarrays applied to the ionizing radiation response. Proc Natl Acad Sci U S A.

[B59] Livak KJ, Schmittgen TD (2001). Analysis of relative gene expression data using real-time quantitative PCR and the 2(-Delta Delta C(T)) Method. Methods.

