# Supplementary material for: Innate gene repression associated with Mycobacterium bovis infection in cattle: toward a gene signature of disease
Source: BMC Genomics. 2007 Oct 31;8:400. doi: 10.1186/1471-2164-8-400 (PMC2213678; doi:10.1186/1471-2164-8-400)
Supplement: Additional file 1 — BOTL-5 microarray spot features that showed significant differential expression between the BTB-infected and non-infected control animals. 378 BOTL-5 microarray spot features that showed significant differential expression between the BTB-infected and non-infected control animals at the P ≤ 0.05 level. [file 1471-2164-8-400-S1.pdf]

Paper Title: Innate gene repression associated with *Mycobacterium bovis* infection in cattle: toward a gene signature of disease. Meade, K.G. *et al.*

Table S1: 378 BOTL-5 microarray spot features that showed significant differential expression between the BTB-infected and non-infected control animals at the $P \leq 0.05$ level. Spot features are ranked by *P*-value and the fold-change for each gene is expressed as the BTB-infected group versus the control group. [NB. light red shading indicates significant differential expression at the $P \leq 0.01$ level]

Rank	BOTL-5 coordinates	Feature description	Gene symbol	*P*-value	Fold-change
1	B5.a7	BOTL0100004XD01R		0.0000019	-1.41
2	A11.f7	zinc finger, DHHC-type containing 19	*ZDHHC19*	0.0000138	1.94
3	D4.b7	BOTL0100013_F01		0.0001301	-1.50
4	B9.h9	nuclear receptor co-repressor 1	*NCOR1*	0.0002447	-2.24
5	B11.e3	G protein-coupled receptor 98	*GPR98*	0.0003538	2.08
6	D2.d8	TANK-binding kinase 1	*TBK1*	0.0004030	-1.75
7	A4.e9	splicing factor proline/glutamine-rich (polypyrimidine tract binding protein associated)	*SFPQ*	0.0004477	-1.73
8	D5.a3	BOTL0100003XF01R		0.0004732	-2.19
9	A4.e3	28S_ribosomal_RNA_gene		0.0004790	1.58
10	D6.i2	neuropilin 1	*NRP1*	0.0005190	-3.18
11	D5.e8	BOTL0100003XF01R		0.0005390	-2.37
12	B9.d8	nuclear receptor co-repressor 1	*NCOR1*	0.0005404	-2.01
13	A7.a5	uncoupling protein 2 (mitochondrial, proton carrier)	*UCP2*	0.0005476	-1.83
14	A1.a7	BOTL0100002XD04R		0.0006579	-1.51

Rank	BOTL-5 coordinates	Feature description	Gene symbol	*P*-value	Fold-change
15	D2.i2	fibroblast growth factor receptor 1 (fms-related tyrosine kinase 2, Pfeiffer syndrome)	*FGFR1*	0.0008729	-2.82
16	A10.f5	nurim (nuclear envelope membrane protein)	*NRM*	0.0009069	-3.47
17	C6.i2	protein phosphatase 2, regulatory subunit B', beta isoform	*PPP2R5B*	0.0009699	1.49
18	A12.c4	HD domain containing 2	*HDDC2*	0.0010459	1.93
19	A8.f5	BOTL0100006XH06R		0.0012679	-3.30
20	A6.b9	hypothetical LOC506974	*LOC506974*	0.0013024	-1.46
21	C11.c1	BTG family, member 3	*BTG3*	0.0013132	-1.46
22	D8.g1	host cell factor C1 (VP16-accessory protein)	*HCFC1*	0.0013267	-2.08
23	A11.a3	glioma tumor suppressor candidate region gene 2	*GLTSCR2*	0.0014304	-2.07
24	C12.g8	BOTL0100013_D12		0.0015900	1.46
25	D1.e4	GATA binding protein 4	*GATA4*	0.0016028	-2.85
26	D1.i6	GATA binding protein 4	*GATA4*	0.0016074	-2.68
27	D2.d9	fibroblast growth factor receptor 1 (fms-related tyrosine kinase 2, Pfeiffer syndrome)	*FGFR1*	0.0016083	-2.73
28	B2.b5	translocation associated membrane protein 1	*TRAM1*	0.0016721	-3.11
29	D9.d7	caspase 10, apoptosis-related cysteine peptidase	*CASP10*	0.0017394	1.38
30	B9.f3	transportin 2 (importin 3, karyopherin beta 2b)	*TNPO2*	0.0017477	1.91
31	B9.i3	v-akt murine thymoma viral oncogene homolog 1	*AKT1*	0.0017768	-2.77
32	C6.g9	colony stimulating factor 2 receptor, alpha, low-affinity (granulocyte-macrophage)	*CSF2RA*	0.0019884	-3.61
33	D5.e1	prohibitin 2	*PHB2*	0.0020928	-1.93

Rank	BOTL-5 coordinates	Feature description	Gene symbol	*P*-value	Fold-change
34	D11.i2	ribosomal protein S6 kinase, 70kDa, polypeptide 2	*RPS6KB2*	0.0021206	-2.50
35	D4.g1	BOTL0100013_F01		0.0021400	-1.67
36	D6.d1	myeloid cell leukemia sequence 1 (BCL2-related)	*MCL1*	0.0021593	-2.14
37	D6.f1	DAZ associated protein 2	*DAZAP2*	0.0021844	-2.70
38	D5.g5	BOTL0100011_F08		0.0023427	1.59
39	A11.e9	major histocompatibility complex, class II, DR alpha	*HLA-DRA*	0.0023432	-1.75
40	C12.b8	transformer-2 alpha	*TRA2A*	0.0023524	-1.90
41	D8.g3	Ran GTPase activating protein 1	*RANGAP1*	0.0023614	-1.45
42	B4.b5	BOTL0100008_H02		0.0023660	-1.66
43	D6.g2	BOTL0100013_F12		0.0023730	-2.33
44	A6.a4	nudix (nucleoside diphosphate linked moiety X)-type motif 8	*NUDT8*	0.0024655	-1.16
45	D6.d9	neuropilin 1	*NRP1*	0.0024810	-2.77
46	B9.c2	BOTL0100011_H03		0.0025029	1.53
47	D8.b7	host cell factor C1 (VP16-accessory protein)	*HCFC1*	0.0025433	-2.08
48	A9.f8	BOTL0100003XA12R		0.0026493	1.61
49	A2.b5	mitogen-activated protein kinase kinase kinase kinase 2	*MAP4K2*	0.0026642	-1.65
50	A12.g6	HD domain containing 2	*HDDC2*	0.0027764	1.71
51	B2.f8	translocation associated membrane protein 1	*TRAM1*	0.0028550	-3.03
52	A6.g3	hypothetical LOC506974	*LOC506974*	0.0028593	-1.49

Rank	BOTL-5 coordinates	Feature description	Gene symbol	*P*-value	Fold-change
53	D11.d9	ribosomal protein S6 kinase, 70kDa, polypeptide 2	*RPS6KB2*	0.0028639	-2.32
54	C10.g4	BOTL0100013_C02		0.0028741	1.26
55	A12.b7	BOTL0100012_F09		0.0029204	1.68
56	A7.f1	uncoupling protein 2 (mitochondrial, proton carrier)	*UCP2*	0.0029412	-1.78
57	B6.i2	nuclear factor of kappa light polypeptide gene enhancer in B-cells inhibitor, zeta	*NFKBIZ*	0.0029766	-3.03
58	D6.b8	BOTL0100013_F12		0.0030079	-2.23
59	C6.c6	colony stimulating factor 2 receptor, alpha, low-affinity (granulocyte-macrophage)	*CSF2RA*	0.0030646	-3.74
60	A11.e8	glioma tumor suppressor candidate region gene 2	*GLTSCR2*	0.0030666	-2.26
61	C1.g6	mitogen-activated protein kinase kinase 7	*MAP2K7*	0.0031151	-2.57
62	C8.e2	SMAD family member 7	*SMAD7*	0.0031160	-1.54
63	A11.a4	major histocompatibility complex, class II, DR alpha	*HLA-DRA*	0.0031203	-1.62
64	A4.i5	28S_ribosomal_RNA_gene		0.0032932	1.46
65	D5.c2	BOTL0100011_F08		0.0033658	1.53
66	C4.a4	mannosyl (alpha-1,6-)-glycoprotein beta-1,2-N-acetylglucosaminyltransferase	*MGAT2*	0.0034894	-1.53
67	C12.g2	transformer-2 alpha	*TRA2A*	0.0034953	-2.01
68	D6.a5	DAZ associated protein 2	*DAZAP2*	0.0035257	-2.40
69	B3.e3	chemokine (C-C motif) receptor 7	*CCR7*	0.0035324	-2.40
70	A4.a4	splicing factor proline/glutamine-rich (polypyrimidine tract binding protein associated)	*SFPQ*	0.0036285	-1.62
71	D12.c9	actin, beta	*ACTB*	0.0036599	-2.60

Rank	BOTL-5 coordinates	Feature description	Gene symbol	*P*-value	Fold-change
72	C9.e2	Mdm2, transformed 3T3 cell double minute 2, p53 binding protein (mouse)	*MDM2*	0.0037452	-1.31
73	D6.b5	BOTL0100009_A01		0.0037458	-1.51
74	C7.f7	BOTL0100002XH09R		0.0037902	-2.12
75	C4.e9	mannosyl (alpha-1,6-)-glycoprotein beta-1,2-N-acetylglucosaminyltransferase	*MGAT2*	0.0039099	-1.50
76	D9.e7	BF076261_cAMP-dependent_protein_kinase_RI-beta		0.0039107	-2.28
77	B12.a5	immediate early response 5	*IER5*	0.0039860	-1.53
78	A4.b9	ELL associated factor 1	*EAF1*	0.0039927	-1.34
79	C7.a8	osteoglycin	*OGN*	0.0039958	1.58
80	B4.f8	BOTL0100008_H02		0.0041364	-1.60
81	D6.i3	CASP8 and FADD-like apoptosis regulator	*CFLAR*	0.0042129	-1.71
82	C2.c1	TAF6 RNA polymerase II, TATA box binding protein (TBP)-associated factor, 80kDa	*TAF6*	0.0042483	-2.34
83	B2.d9	fibroblast growth factor receptor 2 (bacteria-expressed kinase, keratinocyte growth factor receptor, craniofacial dysostosis 1, Crouzon syndrome, Pfeiffer syndrome, Jackson-Weiss syndrome)	*FGFR2*	0.0042637	-1.98
84	C7.f5	osteoglycin	*OGN*	0.0043193	1.48
85	B9.e1	v-akt murine thymoma viral oncogene homolog 1	*AKT1*	0.0043350	-2.64
86	A6.g2	SET binding factor 1	*SBF1*	0.0043798	-1.90
87	C6.h7	nuclear factor of kappa light polypeptide gene enhancer in B-cells 1 (p105)	*NFKB1*	0.0044847	-2.73
88	D11.e6	jun D proto-oncogene	*JUND*	0.0045026	1.40
89	B10.f3	elongation factor RNA polymerase II-like 3	*ELL3*	0.0046062	1.51

Rank	BOTL-5 coordinates	Feature description	Gene symbol	*P*-value	Fold-change
90	A9.g4	BOTL0100010_D08		0.0046436	1.59
91	A9.g5	component of oligomeric golgi complex 4	*COG4*	0.0046664	1.61
92	B9.a3	BOTL0100003XG08R		0.0046742	-1.71
93	B6.g2	echinoderm microtubule associated protein like 2	*EML2*	0.0048091	-2.13
94	D10.e8	tyrosine 3-monooxygenase/tryptophan 5-monooxygenase activation protein, gamma polypeptide	*YWHAG*	0.0048779	1.72
95	A9.a3	tyrosine 3-monooxygenase/tryptophan 5-monooxygenase activation protein, eta polypeptide	*YWHAH*	0.0048914	1.60
96	B9.b9	BOTL0100011_C03		0.0050056	1.71
97	C5.c4	Notch homolog 2 (Drosophila)	*NOTCH2*	0.0050484	-1.58
98	C5.g5	BOTL0100010_C03		0.0050628	-2.22
99	A12.f3	suppressor of Ty 3 homolog (S. cerevisiae)	*SUPT3H*	0.0051309	1.70
100	C3.g3	BOTL0100009_E08		0.0054393	1.76
101	C3.f9	BOTL0100009_D12		0.0058036	1.44
102	D6.h4	myeloid cell leukemia sequence 1 (BCL2-related)	*MCL1*	0.0058114	-2.37
103	B11.i5	G protein-coupled receptor 98	*GPR98*	0.0061638	2.07
104	A9.f5	BOTL0100003XA07R		0.0061795	-1.72
105	C5.i3	macrophage migration inhibitory factor (glycosylation-inhibiting factor)	*MIF*	0.0061871	-2.07
106	C12.i4	coagulation_factor_VII		0.0062506	1.52
107	C8.f8	nurim (nuclear envelope membrane protein)	*NRM*	0.0062870	1.66
108	B9.a7	transportin 2 (importin 3, karyopherin beta 2b)	*TNPO2*	0.0063557	1.35

Rank	BOTL-5 coordinates	Feature description	Gene symbol	*P*-value	Fold-change
109	A9.e9	serine dehydratase	*SDS*	0.0064898	-2.63
110	B6.b8	echinoderm microtubule associated protein like 2	*EML2*	0.0065350	-1.99
111	D11.g6	BOTL0100012_A06		0.0065598	1.38
112	D7.f8	ADP-ribosyltransferase (NAD+) poly (ADP-ribose) polymerase)	*ADPRT*	0.0066591	1.24
113	D4.f9	Fanconi anemia, complementation group A	*FANCA*	0.0066791	-1.40
114	B3.i5	chemokine (C-C motif) receptor 7	*CCR7*	0.0067403	-2.59
115	D5.h5	serine/threonine kinase 17b	*STK17B*	0.0068777	-2.42
116	B4.h7	myeloproliferative leukemia virus oncogene	*MPL*	0.0068954	1.77
117	A12.f8	BOTL0100008_A03		0.0070473	1.50
118	A1.f3	BOTL0100002XD04R		0.0070696	-1.64
119	A3.c7	CD34 molecule	*CD34*	0.0070750	1.74
120	C5.c2	BOTL0100010_C03		0.0070760	-2.10
121	C8.c6	chemokine (C-C motif) ligand 1	*CCL1*	0.0071459	-4.95
122	A6.b8	SET binding factor 1	*SBF1*	0.0072729	-1.77
123	D5.i3	prohibitin 2	*PHB2*	0.0073849	-1.86
124	D11.e1	mitogen-activated protein kinase kinase kinase 11	*MAP3K11*	0.0074285	-3.28
125	A2.b8	family with sequence similarity 125, member A	*FAM125A*	0.0075258	-1.54
126	A5.g4	ubiquitin specific peptidase 3	*USP3*	0.0078067	-1.29
127	A4.g4	zinc finger protein 250	*ZNF250*	0.0078097	-1.43

Rank	BOTL-5 coordinates	Feature description	Gene symbol	*P*-value	Fold-change
128	B4.f7	glutathione synthetase	*GSS*	0.0078180	-1.71
129	A5.g1	actin-bundling protein with BAIAP2 homology	*ABBA-1*	0.0078472	-1.43
130	C1.c4	mitogen-activated protein kinase kinase 7	*MAP2K7*	0.0079342	-2.66
131	B1.h9	Rho guanine nucleotide exchange factor (GEF) 1	*ARHGEF1*	0.0081270	-2.01
132	D8.b9	Ran GTPase activating protein 1	*RANGAP1*	0.0082155	-1.41
133	D8.e2	GCN5 general control of amino-acid synthesis 5-like 2 (yeast)	*GCN5L2*	0.0082224	-1.44
134	B6.d9	nuclear factor of kappa light polypeptide gene enhancer in B-cells inhibitor, zeta	*NFKBIZ*	0.0082446	-2.83
135	A4.i8	cyclin-dependent kinase inhibitor 1A (p21, Cip1)	*CDKN1A*	0.0083509	-2.25
136	A8.g9	major histocompatibility complex, class I, A	*HLA-A*	0.0084270	-5.13
137	C4.i8	AXL receptor tyrosine kinase	*AXL*	0.0085442	1.59
138	B9.e2	C-terminal binding protein 1	*CTBP1*	0.0087092	-2.35
139	C10.h2	proprotein convertase subtilisin/kexin type 6	*PCSK6*	0.0087853	1.73
140	C9.i4	Mdm2, transformed 3T3 cell double minute 2, p53 binding protein (mouse)	*MDM2*	0.0090137	-1.48
141	B1.d8	Rho guanine nucleotide exchange factor (GEF) 1	*ARHGEF1*	0.0090614	-1.74
142	D12.h3	actin, beta	*ACTB*	0.0091130	-2.42
143	A4.g3	ELL associated factor 1	*EAF1*	0.0091441	-1.26
144	B5.g4	BOTL0100011_F05		0.0091696	1.72
145	B11.e8	hydroxysteroid (17-beta) dehydrogenase 4	*HSD17B4*	0.0093751	1.45
146	C5.e8	tropomyosin 3	*TPM3*	0.0093914	-2.26

Rank	BOTL-5 coordinates	Feature description	Gene symbol	*P*-value	Fold-change
147	A6.f7	poly (ADP-ribose) polymerase family, member 10	*PARP10*	0.0095845	-1.34
148	D9.i6	v-akt murine thymoma viral oncogene homolog 2	*AKT2*	0.0096285	-2.42
149	D2.h9	TANK-binding kinase 1	*TBK1*	0.0096930	-1.50
150	C8.g9	chemokine (C-C motif) ligand 1	*CCL1*	0.0098124	-4.78
151	C10.e1	nuclear factor of activated T-cells, cytoplasmic, calcineurin-dependent 4	*NFATC4*	0.0099192	1.54
152	C6.b9	transcription elongation factor A (SII)-like 4	*TCEAL4*	0.0100420	1.33
153	B2.g9	bone morphogenetic protein 4	*BMP4*	0.0100947	-4.73
154	C2.i4	retinoid X receptor, beta	*RXRB*	0.0101659	-1.43
155	B6.e2	eukaryotic translation elongation factor 2	*EEF2*	0.0102301	-2.29
156	B2.b9	immediate early response 5	*IER5*	0.0102766	-1.70
157	A4.e7	cyclin-dependent kinase inhibitor 1A (p21, Cip1)	*CDKN1A*	0.0103707	-2.20
158	D7.a3	DCN1, defective in cullin neddylation 1, domain containing 5 (S. cerevisiae)	*DCUN1D5*	0.0104958	1.51
159	A1.c1	BOTL0100009_H11		0.0106842	-1.45
160	D1.e6	serum response factor (c-fos serum response element-binding transcription factor)	*SRF*	0.0108486	-1.91
161	B6.i4	eukaryotic translation elongation factor 2	*EEF2*	0.0109193	-2.26
162	C6.d7	nuclear factor of kappa light polypeptide gene enhancer in B-cells 1 (p105)	*NFKB1*	0.0109787	-3.05
163	C9.b7	eukaryotic translation initiation factor 5	*EIF5*	0.0110766	-1.88
164	B1.e6	GATA binding protein 4	*GATA4*	0.0110811	-2.62
165	C7.b3	BOTL0100002XH09R		0.0113914	-1.92

Rank	BOTL-5 coordinates	Feature description	Gene symbol	*P*-value	Fold-change
166	B4.d7	myeloproliferative leukemia virus oncogene	*MPL*	0.0115917	1.71
167	A9.a4	serine dehydratase	*SDS*	0.0120906	-2.46
168	B5.d8	SMAD family member 2	*SMAD2*	0.0123800	1.42
169	D11.i3	mitogen-activated protein kinase kinase kinase 11	*MAP3K11*	0.0125362	-3.29
170	C8.i4	SMAD family member 7	*SMAD7*	0.0126812	-1.45
171	A12.f7	BOTL0100007_G05		0.0129730	1.73
172	B2.g3	immediate early response 5	*IER5*	0.0129934	-1.77
173	B5.c1	BOTL0100011_F05		0.0132287	1.64
174	C6.d9	protein phosphatase 2, regulatory subunit B', beta isoform	*PPP2R5B*	0.0132696	1.38
175	A4.c1	zinc finger protein 250	*ZNF250*	0.0132725	-1.31
176	A9.f3	BOTL0100003XA03R		0.0134318	-1.52
177	C11.g4	BTG family, member 3	*BTG3*	0.0135524	-1.36
178	A10.a8	nurim (nuclear envelope membrane protein)	*NRM*	0.0135772	-2.67
179	A9.a6	proteasome (prosome, macropain) 26S subunit, non-ATPase, 13	*PSMD13*	0.0136414	1.27
180	C6.b5	BOTL0100006XH02R		0.0136797	1.61
181	D6.i4	CAM_kinase_I		0.0138000	-1.48
182	A9.b8	chromosome 2 open reading frame 25	*C2orf25*	0.0140830	1.55
183	A6.e3	AXL receptor tyrosine kinase	*AXL*	0.0141779	1.65
184	A12.g1	BOTL0100012_F09		0.0143107	1.69

Rank	BOTL-5 coordinates	Feature description	Gene symbol	*P*-value	Fold-change
185	B11.c2	similar to Succinyl-CoA ligase [GDP-forming] beta-chain, mitochondrial precursor (Succinyl-CoA synthetase, betaG chain) (SCS-betaG) (GTP-specific succinyl-CoA synthetase beta subunit)	*LOC283398*	0.0149054	1.61
186	D9.h7	caspase 10, apoptosis-related cysteine peptidase	*CASP10*	0.0149277	1.42
187	C11.f8	similar to PRIP-interacting protein PIPMT	*LOC511392*	0.0149358	1.37
188	C8.a4	Wiskott-Aldrich syndrome (eczema-thrombocytopenia)	*WAS*	0.0153904	-1.64
189	D9.i2	v-akt murine thymoma viral oncogene homolog 1	*AKT1*	0.0154488	-2.23
190	B7.h9	formin-like 3	*FMNL3*	0.0158468	-2.56
191	D8.h7	platelet-derived growth factor beta polypeptide (simian sarcoma viral (v-sis) oncogene homolog)	*PDGFB*	0.0158677	1.65
192	C3.b6	BOTL0100009_D12		0.0161245	1.45
193	B8.b7	prohibitin 2	*PHB2*	0.0161357	-1.34
194	B5.c4	BOTL0100011_F09		0.0163650	1.54
195	C12.c1	peroxisomal biogenesis factor 16	*PEX16*	0.0163872	1.55
196	B12.a4	serine dehydratase	*SDS*	0.0164980	-1.90
197	C10.g8	similar to leukocyte differentiation antigen CD84	*LOC510910*	0.0165416	1.56
198	C11.g1	BOTL0100009_H06		0.0166329	-1.92
199	D4.b6	Fanconi anemia, complementation group A	*FANCA*	0.0166410	-1.37
200	B8.b3	BOTL0100009_A07		0.0169136	1.60
201	A4.f7	BOTL0100006XF10R		0.0169253	-1.22
202	C2.e4	growth arrest and DNA-damage-inducible, alpha	*GADD45A*	0.0170333	1.55

Rank	BOTL-5 coordinates	Feature description	Gene symbol	*P*-value	Fold-change
203	C11.f7	bromodomain containing 2	*BRD2*	0.0170426	-1.55
204	D2.a3	Cbp/p300-interacting transactivator, with Glu/Asp-rich carboxy-terminal domain, 2	*CITED2*	0.0170796	-1.47
205	D9.i8	BF076261_cAMP-dependent_protein_kinase_RI-beta		0.0170940	-2.14
206	D8.h6	endothelial cell growth factor 1 (platelet-derived)	*ECGF1*	0.0173043	1.79
207	B1.i7	GATA binding protein 4	*GATA4*	0.0173044	-2.98
208	B7.d8	formin-like 3	*FMNL3*	0.0173690	-2.57
209	C6.h1	melanin-concentrating hormone receptor 1	*MCHR1*	0.0174139	1.79
210	A5.a3	glyceraldehyde-3-phosphate dehydrogenase	*GAPDH*	0.0174737	-1.43
211	B10.e9	transducin (beta)-like 1X-linked	*TBL1X*	0.0176459	1.54
212	A8.g2	BOTL0100012_E05		0.0178975	-1.48
213	B5.i4	growth hormone 2	*GH2*	0.0179332	-1.59
214	A3.c5	immediate early response 5	*IER5*	0.0179898	-1.27
215	D9.e4	v-akt murine thymoma viral oncogene homolog 2	*AKT2*	0.0179901	-2.40
216	C12.g3	BOTL0100012_G03		0.0181028	1.30
217	D5.f3	exosome component 6	*EXOSC6*	0.0185682	-1.30
218	B8.d7	platelet-derived growth factor alpha polypeptide	*PDGFA*	0.0190129	1.73
219	B8.g1	prohibitin 2	*PHB2*	0.0193058	-1.47
220	B2.i2	fibroblast growth factor receptor 2 (bacteria-expressed kinase, keratinocyte growth factor receptor, craniofacial dysostosis 1, Crouzon syndrome, Pfeiffer syndrome, Jackson-Weiss syndrome)	*FGFR2*	0.0193394	-2.06

Rank	BOTL-5 coordinates	Feature description	Gene symbol	*P*-value	Fold-change
221	A3.c6	CD14 molecule	*CD14*	0.0194294	-3.43
222	C11.c2	lysosomal associated multispanning membrane protein 5	*LAPTM5*	0.0194488	-3.13
223	B8.e1	RAS p21 protein activator 3	*RASA3*	0.0195049	-1.83
224	B4.b3	glutathione synthetase	*GSS*	0.0196135	-1.65
225	C2.g4	TAF6 RNA polymerase II, TATA box binding protein (TBP)-associated factor, 80kDa	*TAF6*	0.0196589	-2.15
226	B2.c6	bone morphogenetic protein 4	*BMP4*	0.0197244	-3.26
227	C11.a3	zinc finger protein 786	*ZNF786*	0.0202787	1.29
228	A4.h9	conserved helix-loop-helix ubiquitous kinase	*CHUK*	0.0203143	-2.12
229	B3.h9	EPH receptor B1	*EPHB1*	0.0204831	-1.74
230	A8.g3	BOTL0100012_E08		0.0206837	-1.29
231	D10.f8	LOC515347		0.0208392	-1.29
232	C2.h9	protein kinase C, beta 1	*PRKCB1*	0.0210312	-1.50
233	B9.e8	BOTL0100003XG08R		0.0210765	-1.56
234	C11.c7	interleukin 16 (lymphocyte chemoattractant factor)	*IL16*	0.0211118	1.79
235	B3.f5	BOTL0100004XC03R		0.0213766	1.59
236	C11.g5	lysosomal associated multispanning membrane protein 5	*LAPTM5*	0.0217915	-3.17
237	C5.e1	macrophage migration inhibitory factor (glycosylation-inhibiting factor)	*MIF*	0.0218428	-1.74
238	D10.f7	malignant T cell amplified sequence 1	*MCTS1*	0.0219207	1.54
239	B6.a5	BOTL0100008_C07		0.0221897	1.58

Rank	BOTL-5 coordinates	Feature description	Gene symbol	*P*-value	Fold-change
240	B9.g3	BOTL0100011_C03		0.0224630	1.60
241	A9.a8	BOTL0100003XA07R		0.0225451	-1.58
242	B6.i3	eukaryotic translation elongation factor 1 gamma	*EEF1G*	0.0227883	-2.08
243	D8.d5	endothelial cell growth factor 1 (platelet-derived)	*ECGF1*	0.0229457	1.75
244	C12.a4	BOTL0100006XD09R		0.0235544	1.51
245	D10.e9	BOTL0100008_E02		0.0236260	-1.73
246	C10.c8	proprotein convertase subtilisin/kexin type 6	*PCSK6*	0.0240355	1.73
247	C4.i2	inhibitor of kappa light polypeptide gene enhancer in B-cells, kinase beta	*IKBKB*	0.0241572	-1.70
248	C10.b7	similar to cell death regulator aven	*LOC512666*	0.0242998	1.63
249	B3.d9	myelin basic protein	*MBP*	0.0244414	-2.95
250	A1.c5	endonuclease domain containing 1	*ENDOD1*	0.0245069	-1.57
251	D7.b9	integrin-linked kinase-associated serine/threonine phosphatase 2C	*ILKAP*	0.0246783	1.38
252	A4.d8	conserved helix-loop-helix ubiquitous kinase	*CHUK*	0.0250975	-1.74
253	A2.b3	zinc finger protein 324	*ZNF324*	0.0257520	-1.33
254	A5.b7	actin-bundling protein with BAIAP2 homology	*ABBA-1*	0.0258944	-1.37
255	B8.h7	platelet-derived growth factor alpha polypeptide	*PDGFA*	0.0262092	1.68
256	C8.e9	Wiskott-Aldrich syndrome (eczema-thrombocytopenia)	*WAS*	0.0265945	-1.57
257	B5.a3	protein tyrosine phosphatase, receptor type, C-associated protein	*PTPRCAP*	0.0266610	1.54
258	D11.a7	tripartite motif-containing 13	*TRIM13*	0.0269797	1.44

Rank	BOTL-5 coordinates	Feature description	Gene symbol	*P*-value	Fold-change
259	A3.b6	BOTL0100009_D11		0.0270896	-1.60
260	D10.b7	methyltransferase like 5	*METTL5*	0.0273635	1.44
261	D6.e1	CASP8 and FADD-like apoptosis regulator	*CFLAR*	0.0273795	-1.72
262	D3.f1	zinc finger, DHHC-type containing 7	*ZDHHC7*	0.0275724	-1.59
263	B9.f7	BOTL0100004XG08R		0.0282106	1.37
264	C1.b9	vacuolar protein sorting 13 homolog C (S. cerevisiae)	*VPS13C*	0.0284981	1.60
265	B7.e7	mitogen-activated protein kinase kinase 1	*MAP2K1*	0.0286397	-1.48
266	A10.i4	Rap guanine nucleotide exchange factor (GEF) 1	*RAPGEF1*	0.0286460	1.68
267	A3.b3	F-box and leucine-rich repeat protein 11	*FBXL11*	0.0289069	-1.58
268	B2.a6	jun oncogene	*JUN*	0.0289247	1.47
269	A6.i5	AXL receptor tyrosine kinase	*AXL*	0.0290260	1.52
270	C10.a4	poly (ADP-ribose) polymerase family, member 10	*PARP10*	0.0290333	1.52
271	B6.b6	BOTL0100013_F06		0.0292628	-1.20
272	B8.i3	RAS p21 protein activator 3	*RASA3*	0.0292993	-1.82
273	D3.i5	MAP kinase interacting serine/threonine kinase 1	*MKNK1*	0.0294027	-1.77
274	A11.f8	alanyl-tRNA synthetase domain containing 1	*AARSD1*	0.0295263	1.19
275	C9.c2	BOTL0100010_D12		0.0299158	1.79
276	D9.f9	BOTL0100011_B08		0.0301768	-1.53
277	C7.g4	neurobeachin-like 2	*NBEAL2*	0.0302027	-1.34

Rank	BOTL-5 coordinates	Feature description	Gene symbol	*P*-value	Fold-change
278	C6.e3	matrix metallopeptidase 28	*MMP28*	0.0302195	1.23
279	C2.i3	NBFGC_AW357604		0.0303135	-1.79
280	B6.e1	eukaryotic translation elongation factor 1 gamma	*EEF1G*	0.0304441	-2.07
281	B2.e8	chromosome 12 open reading frame 35	*C12orf35*	0.0309394	-1.92
282	D3.a5	zinc finger, DHHC-type containing 7	*ZDHHC7*	0.0309508	-1.49
283	D3.e3	MAP kinase interacting serine/threonine kinase 1	*MKNK1*	0.0311134	-1.97
284	C11.b5	similar to PRIP-interacting protein PIPMT	*LOC511392*	0.0312712	1.30
285	C11.b3	bromodomain containing 2	*BRD2*	0.0313898	-1.80
286	B9.i4	C-terminal binding protein 1	*CTBP1*	0.0315112	-2.23
287	B9.e4	secreted frizzled-related protein 1	*SFRP1*	0.0316679	-1.87
288	C10.b6	autocrine motility factor receptor	*AMFR*	0.0317547	1.35
289	B9.f5	BOTL0100004XG06R		0.0318870	1.66
290	D2.e8	Cbp/p300-interacting transactivator, with Glu/Asp-rich carboxy-terminal domain, 2	*CITED2*	0.0320792	-1.48
291	D8.d7	platelet-derived growth factor beta polypeptide (simian sarcoma viral (v-sis) oncogene homolog)	*PDGFB*	0.0321179	1.57
292	A10.b3	insulin induced gene 1	*INSIG1*	0.0321826	1.43
293	B4.d8	growth hormone receptor	*GHR*	0.0324739	-1.70
294	C12.c5	BOTL0100013_D12		0.0326116	1.77
295	B5.i2	BRCA1 associated protein-1 (ubiquitin carboxy-terminal hydrolase)	*BAP1*	0.0326501	-2.10
296	D8.b8	nucleoporin 62kDa	*NUP62*	0.0326634	-1.37

Rank	BOTL-5 coordinates	Feature description	Gene symbol	*P*-value	Fold-change
297	C6.b7	chromosome 3 open reading frame 28	*C3orf28*	0.0328473	1.24
298	A9.c1	BOTL0100010_D08		0.0330704	1.58
299	B9.f1	zinc finger, DHHC-type containing 7	*ZDHHC7*	0.0331515	1.45
300	A6.e9	nudix (nucleoside diphosphate linked moiety X)-type motif 8	*NUDT8*	0.0332678	-1.17
301	B11.a3	hydroxysteroid (17-beta) dehydrogenase 4	*HSD17B4*	0.0333190	1.35
302	A8.c4	BOTL0100013_B08		0.0334421	1.63
303	A5.i4	nuclear receptor co-repressor 1	*NCOR1*	0.0335313	-1.74
304	D5.h9	casein kinase 1, gamma 1	*CSNK1G1*	0.0336361	-1.38
305	A4.e1	ELK1, member of ETS oncogene family	*ELK1*	0.0337790	1.58
306	C9.g1	eukaryotic translation initiation factor 5	*EIF5*	0.0346893	-1.74
307	B5.b9	BOTL0100011_A03		0.0347966	-1.24
308	C11.b7	BOTL0100009_H06		0.0357204	-1.84
309	C5.a3	tropomyosin 3	*TPM3*	0.0357426	-2.00
310	B3.e4	phosphoinositide-3-kinase, regulatory subunit 5, p101	*PIK3R5*	0.0358222	-1.41
311	B12.f1	immediate early response 5	*IER5*	0.0359498	-1.38
312	D10.a4	BOTL0100008_E02		0.0362718	-2.27
313	A3.g4	protein tyrosine phosphatase, receptor type, C-associated protein	*PTPRCAP*	0.0363632	-1.44
314	A11.d3	tumor necrosis factor (TNF superfamily, member 2)	*TNF*	0.0370094	1.72
315	C6.g3	transcription elongation factor A (SII)-like 4	*TCEAL4*	0.0370660	1.37

Rank	BOTL-5 coordinates	Feature description	Gene symbol	*P*-value	Fold-change
316	C10.c5	similar to leukocyte differentiation antigen CD84	*LOC510910*	0.0375443	1.50
317	B3.i2	myelin basic protein	*MBP*	0.0377252	-2.66
318	A7.a3	TBC1 domain family, member 20	*TBC1D20*	0.0377384	-1.37
319	C8.f9	similar to Poly [ADP-ribose] polymerase 10 (PARP-10)	*LOC510991*	0.0378274	1.51
320	C12.b9	BOTL0100012_G03		0.0378284	1.53
321	B7.a8	thioredoxin-like 2	*TXNL2*	0.0378491	1.33
322	C4.e2	protein_kinase_C,_alpha_binding_protein		0.0381032	-1.92
323	D7.f1	BOTL0100003XG05R		0.0381954	-1.31
324	A7.c1	BOTL0100010_C09		0.0382428	1.33
325	C4.g3	triggering receptor expressed on myeloid cells-1	*TREM-1*	0.0385547	-1.71
326	A3.g8	immediate early response 5	*IER5*	0.0385969	-1.27
327	B2.a3	chromosome 12 open reading frame 35	*C12orf35*	0.0387424	-1.62
328	C6.b8	BOTL0100012_D04		0.0394158	-1.76
329	D10.g1	methyltransferase like 5	*METTL5*	0.0395425	1.42
330	D9.f3	tetraspanin 14	*TSPAN14*	0.0395769	1.48
331	B7.a3	BOTL0100003XF12R		0.0398906	1.50
332	B1.e3	heat shock 27kDa protein 1	*HSPB1*	0.0399787	-1.59
333	D2.b8	protein tyrosine phosphatase, receptor type, C-associated protein	*PTPRCAP*	0.0400525	-1.24
334	C9.g5	BOTL0100010_D12		0.0404501	1.42

Rank	BOTL-5 coordinates	Feature description	Gene symbol	*P*-value	Fold-change
335	C11.c5	signal transducer and activator of transcription 6, interleukin-4 induced	*STAT6*	0.0406154	-1.54
336	A11.f2	similar to Lymphocyte antigen Ly-6E precursor (Retinoic acid-induced gene E protein) (RIG-E) (Thymic shared antigen 1) (TSA-1) (Stem cell antigen 2) (SCA-2)	*MGC140297*	0.0410107	-1.64
337	B9.a5	zinc finger, DHHC-type containing 7	*ZDHHC7*	0.0411976	1.38
338	A2.c1	EH domain binding protein 1-like 1	*EHBP1L1*	0.0412845	1.25
339	A3.g9	CD14 molecule	*CD14*	0.0417879	-2.73
340	D11.e2	kinase suppressor of ras 1	*KSR1*	0.0418199	-2.19
341	B1.b5	cathepsin W	*CTSW*	0.0418369	-1.34
342	B11.b3	cell cycle associated protein 1	*CAPRIN1*	0.0425681	1.43
343	B6.f2	Rho family GTPase 1	*RND1*	0.0427946	-1.82
344	C3.g1	BOTL0100009_E03		0.0430134	1.45
345	D10.b3	malignant T cell amplified sequence 1	*MCTS1*	0.0430731	1.47
346	A11.a6	similar to Lymphocyte antigen Ly-6E precursor (Retinoic acid-induced gene E protein) (RIG-E) (Thymic shared antigen 1) (TSA-1) (Stem cell antigen 2) (SCA-2)	*MGC140297*	0.0431527	-1.62
347	A4.f9	solute carrier family 7 (cationic amino acid transporter, y+ system), member 5	*SLC7A5*	0.0434126	-1.20
348	A2.i3	chemokine (C-C motif) ligand 24	*CCL24*	0.0435507	-1.33
349	C10.b3	BOTL0100007_D05		0.0443755	1.42
350	D6.b7	serine dehydratase-like	*SDSL*	0.0445689	-1.41
351	A9.c4	BOTL0100010_E01		0.0446673	-1.61
352	A8.a8	BOTL0100006XH06R		0.0449694	-2.30

Rank	BOTL-5 coordinates	Feature description	Gene symbol	*P*-value	Fold-change
353	B3.i6	phosphoinositide-3-kinase, regulatory subunit 5, p101	*PIK3R5*	0.0450732	-1.40
354	A2.c4	PX domain containing serine/threonine kinase	*PXK*	0.0451902	-1.45
355	C4.b9	triggering receptor expressed on myeloid cells-1	*TREM-1*	0.0454033	-1.83
356	A8.d3	nerve growth factor receptor (TNFR superfamily, member 16)	*NGFR*	0.0455238	1.64
357	B11.d9	E2F transcription factor 6	*E2F6*	0.0456080	1.43
358	C10.g5	UDP-Gal:betaGlcNAc beta 1,4- galactosyltransferase, polypeptide 2	*B4GALT2*	0.0456436	1.60
359	B8.a6	ring finger protein 145	*RNF145*	0.0458548	1.63
360	D5.d3	serine/threonine kinase 17b	*STK17B*	0.0459282	-1.77
361	A5.f3	eukaryotic translation elongation factor 1 alpha 1	*EEF1A1*	0.0459954	-1.28
362	C3.f3	ribosomal protein, large, P1	*RPLP1*	0.0461129	1.40
363	D6.g1	serine dehydratase-like	*SDSL*	0.0464412	-1.45
364	A8.c6	major histocompatibility complex, class I, A	*HLA-A*	0.0464740	-3.51
365	B3.a6	methionyl-tRNA synthetase	*MARS*	0.0464983	1.81
366	D1.i7	serum response factor (c-fos serum response element-binding transcription factor)	*SRF*	0.0465517	-1.74
367	D10.a3	tyrosine 3-monooxygenase/tryptophan 5-monooxygenase activation protein, gamma polypeptide	*YWHAG*	0.0471314	1.43
368	A8.b7	BOTL0100012_E02		0.0475018	1.71
369	A12.e8	BOTL0100006XD05R		0.0476229	1.46
370	C2.d8	protein kinase C, beta 1	*PRKCB1*	0.0479493	-1.58
371	B9.i8	axin 1	*AXIN1*	0.0481007	-1.40

Rank	BOTL-5 coordinates	Feature description	Gene symbol	*P*-value	Fold-change
372	C6.c7	melanin-concentrating hormone receptor 1	*MCHR1*	0.0487027	1.90
373	B10.a4	transducin (beta)-like 1X-linked	*TBL1X*	0.0489704	1.44
374	A11.g5	adenylate cyclase 7	*ADCY7*	0.0489938	-1.39
375	A6.e1	cadherin 5, type 2, VE-cadherin (vascular epithelium)	*CDH5*	0.0490342	-1.84
376	A8.f8	apolipoprotein B mRNA editing enzyme, catalytic polypeptide-like 3F	*APOBEC3F*	0.0490966	1.66
377	D9.d9	v-akt murine thymoma viral oncogene homolog 1	*AKT1*	0.0496475	-2.28
378	C4.i4	protein_kinase_C,_alpha_binding_protein		0.0498164	-1.86
