# Supplementary material for: Innate gene repression associated with Mycobacterium bovis infection in cattle: toward a gene signature of disease
Source: BMC Genomics. 2007 Oct 31;8:400. doi: 10.1186/1471-2164-8-400 (PMC2213678; doi:10.1186/1471-2164-8-400)
Supplement: Additional file 2 — Genes on the BOTL-5 microarray represented by two or more replicate features that showed significant differential expression between the BTB-infected and non-infected control animals. 122 genes on the BOTL-5 microarray represented by two or more replicate features that showed significant differential expression between the BTB-infected and non-infected control animals at the P ≤ 0.05 level. [file 1471-2164-8-400-S2.pdf]

Paper Title: Innate gene repression associated with *Mycobacterium bovis* infection in cattle: toward a gene signature of disease. Meade, K.G. *et al.*

Table S2: 122 genes on the BOTL-5 microarray represented by two or more replicate features that showed significant differential expression between the BTB-infected and non-infected control animals at the P ≤ 0.05 level. Spot features are ranked by mean fold-change and the fold-changes for each spot feature or gene is expressed as the BTB-infected group versus the control group.

BOTL-5 coordinates	Feature description	Gene symbol	*P*-value	Fold-change	Mean fold-change
C8.c6	chemokine (C-C motif) ligand 1	*CCL1*	0.0071459	-4.95	-4.86
C8.g9	chemokine (C-C motif) ligand 1	*CCL1*	0.0098124	-4.78	
A8.c6	major histocompatibility complex, class I, A	*HLA-A*	0.0464740	-3.51	-4.32
A8.g9	major histocompatibility complex, class I, A	*HLA-A*	0.0084270	-5.13	
B2.c6	bone morphogenetic protein 4	*BMP4*	0.0197244	-3.26	-3.99
B2.g9	bone morphogenetic protein 4	*BMP4*	0.0100947	-4.73	
C6.c6	colony stimulating factor 2 receptor, alpha, low-affinity (granulocyte-macrophage)	*CSF2RA*	0.0030646	-3.74	-3.67
C6.g9	colony stimulating factor 2 receptor, alpha, low-affinity (granulocyte-macrophage)	*CSF2RA*	0.0019884	-3.61	
D11.e1	mitogen-activated protein kinase kinase kinase 11	*MAP3K11*	0.0074285	-3.28	-3.29
D11.i3	mitogen-activated protein kinase kinase kinase 11	*MAP3K11*	0.0125362	-3.29	
C11.c2	lysosomal associated multispanning membrane protein 5	*LAPTM5*	0.0194488	-3.13	-3.15
C11.g5	lysosomal associated multispanning membrane protein 5	*LAPTM5*	0.0217915	-3.17	
A3.c6	CD14 molecule	*CD14*	0.0194294	-3.43	-3.08
A3.g9	CD14 molecule	*CD14*	0.0417879	-2.73	

BOTL-5 coordinates	Feature description	Gene symbol	*P*-value	Fold-change	Mean fold-change
B2.b5	translocation associated membrane protein 1	*TRAM1*	0.0016721	-3.11	-3.07
B2.f8	translocation associated membrane protein 1	*TRAM1*	0.0028550	-3.03	
D6.d9	neuropilin 1	*NRP1*	0.0024810	-2.77	-2.98
D6.i2	neuropilin 1	*NRP1*	0.0005190	-3.18	
B6.d9	nuclear factor of kappa light polypeptide gene enhancer in B-cells inhibitor, zeta	*NFKBIZ*	0.0082446	-2.83	-2.93
B6.i2	nuclear factor of kappa light polypeptide gene enhancer in B-cells inhibitor, zeta	*NFKBIZ*	0.0029766	-3.03	
C6.d7	nuclear factor of kappa light polypeptide gene enhancer in B-cells 1 (p105)	*NFKB1*	0.0109787	-3.05	-2.89
C6.h7	nuclear factor of kappa light polypeptide gene enhancer in B-cells 1 (p105)	*NFKB1*	0.0044847	-2.73	
A8.a8	BOTL0100006XH06R		0.0449694	-2.30	-2.80
A8.f5	BOTL0100006XH06R		0.0012679	-3.30	
B3.d9	myelin basic protein	*MBP*	0.0244414	-2.95	-2.80
B3.i2	myelin basic protein	*MBP*	0.0377252	-2.66	
B1.e6	GATA binding protein 4	*GATA4*	0.0110811	-2.62	-2.78
B1.i7	GATA binding protein 4	*GATA4*	0.0173044	-2.98	
D1.e4	GATA binding protein 4	*GATA4*	0.0016028	-2.85	
D1.i6	GATA binding protein 4	*GATA4*	0.0016074	-2.68	
D2.d9	fibroblast growth factor receptor 1 (fms-related tyrosine kinase 2, Pfeiffer syndrome)	*FGFR1*	0.0016083	-2.73	-2.77
D2.i2	fibroblast growth factor receptor 1 (fms-related tyrosine kinase 2, Pfeiffer syndrome)	*FGFR1*	0.0008729	-2.82	
C1.c4	mitogen-activated protein kinase kinase 7	*MAP2K7*	0.0079342	-2.66	-2.61

BOTL-5 coordinates	Feature description	Gene symbol	*P*-value	Fold-change	Mean fold-change
C1.g6	mitogen-activated protein kinase kinase 7	*MAP2K7*	0.0031151	-2.57	
B7.d8	formin-like 3	*FMNL3*	0.0173690	-2.57	-2.57
B7.h9	formin-like 3	*FMNL3*	0.0158468	-2.56	
D6.a5	DAZ associated protein 2	*DAZAP2*	0.0035257	-2.40	-2.55
D6.f1	DAZ associated protein 2	*DAZAP2*	0.0021844	-2.70	
D12.c9	actin, beta	*ACTB*	0.0036599	-2.60	-2.51
D12.h3	actin, beta	*ACTB*	0.0091130	-2.42	
B3.e3	chemokine (C-C motif) receptor 7	*CCR7*	0.0035324	-2.40	-2.49
B3.i5	chemokine (C-C motif) receptor 7	*CCR7*	0.0067403	-2.59	
B9.e1	v-akt murine thymoma viral oncogene homolog 1	*AKT1*	0.0043350	-2.64	-2.48
B9.i3	v-akt murine thymoma viral oncogene homolog 1	*AKT1*	0.0017768	-2.77	
D9.d9	v-akt murine thymoma viral oncogene homolog 1	*AKT1*	0.0496475	-2.28	
D9.i2	v-akt murine thymoma viral oncogene homolog 1	*AKT1*	0.0154488	-2.23	
D11.d9	ribosomal protein S6 kinase, 70kDa, polypeptide 2	*RPS6KB2*	0.0028639	-2.32	-2.41
D11.i2	ribosomal protein S6 kinase, 70kDa, polypeptide 2	*RPS6KB2*	0.0021206	-2.50	
D9.e4	v-akt murine thymoma viral oncogene homolog 2	*AKT2*	0.0179901	-2.40	-2.41
D9.i6	v-akt murine thymoma viral oncogene homolog 2	*AKT2*	0.0096285	-2.42	
A9.a4	serine dehydratase	*SDS*	0.0120906	-2.46	-2.33
A9.e9	serine dehydratase	*SDS*	0.0064898	-2.63	

BOTL-5 coordinates	Feature description	Gene symbol	*P*-value	Fold-change	Mean fold-change
B12.a4	serine dehydratase	*SDS*	0.0164980	-1.90	
B9.e2	C-terminal binding protein 1	*CTBP1*	0.0087092	-2.35	-2.29
B9.i4	C-terminal binding protein 1	*CTBP1*	0.0315112	-2.23	
D6.b8	BOTL0100013_F12		0.0030079	-2.23	-2.28
D6.g2	BOTL0100013_F12		0.0023730	-2.33	
D5.a3	BOTL0100003XF01R		0.0004732	-2.19	-2.28
D5.e8	BOTL0100003XF01R		0.0005390	-2.37	
B6.e2	eukaryotic translation elongation factor 2	*EEF2*	0.0102301	-2.29	-2.27
B6.i4	eukaryotic translation elongation factor 2	*EEF2*	0.0109193	-2.26	
D6.d1	myeloid cell leukemia sequence 1 (BCL2-related)	*MCL1*	0.0021593	-2.14	-2.25
D6.h4	myeloid cell leukemia sequence 1 (BCL2-related)	*MCL1*	0.0058114	-2.37	
C2.c1	TAF6 RNA polymerase II, TATA box binding protein (TBP)-associated factor, 80kDa	*TAF6*	0.0042483	-2.34	-2.25
C2.g4	TAF6 RNA polymerase II, TATA box binding protein (TBP)-associated factor, 80kDa	*TAF6*	0.0196589	-2.15	
A4.e7	cyclin-dependent kinase inhibitor 1A (p21, Cip1)	*CDKN1A*	0.0103707	-2.20	-2.22
A4.i8	cyclin-dependent kinase inhibitor 1A (p21, Cip1)	*CDKN1A*	0.0083509	-2.25	
D9.e7	BF076261_cAMP-dependent_protein_kinase_RI-beta		0.0039107	-2.28	-2.21
D9.i8	BF076261_cAMP-dependent_protein_kinase_RI-beta		0.0170940	-2.14	
A11.a3	glioma tumor suppressor candidate region gene 2	*GLTSCR2*	0.0014304	-2.07	-2.16
A11.e8	glioma tumor suppressor candidate region gene 2	*GLTSCR2*	0.0030666	-2.26	

BOTL-5 coordinates	Feature description	Gene symbol	*P*-value	Fold-change	Mean fold-change
C5.c2	BOTL0100010_C03		0.0070760	-2.10	-2.16
C5.g5	BOTL0100010_C03		0.0050628	-2.22	
C5.a3	tropomyosin 3	*TPM3*	0.0357426	-2.00	-2.13
C5.e8	tropomyosin 3	*TPM3*	0.0093914	-2.26	
D5.d3	serine/threonine kinase 17b	*STK17B*	0.0459282	-1.77	-2.10
D5.h5	serine/threonine kinase 17b	*STK17B*	0.0068777	-2.42	
D8.b7	host cell factor C1 (VP16-accessory protein)	*HCFC1*	0.0025433	-2.08	-2.08
D8.g1	host cell factor C1 (VP16-accessory protein)	*HCFC1*	0.0013267	-2.08	
B6.e1	eukaryotic translation elongation factor 1 gamma	*EEF1G*	0.0304441	-2.07	-2.07
B6.i3	eukaryotic translation elongation factor 1 gamma	*EEF1G*	0.0227883	-2.08	
B6.b8	echinoderm microtubule associated protein like 2	*EML2*	0.0065350	-1.99	-2.06
B6.g2	echinoderm microtubule associated protein like 2	*EML2*	0.0048091	-2.13	
C7.b3	BOTL0100002XH09R		0.0113914	-1.92	-2.02
C7.f7	BOTL0100002XH09R		0.0037902	-2.12	
B2.d9	fibroblast growth factor receptor 2 (bacteria-expressed kinase, keratinocyte growth factor receptor, craniofacial dysostosis 1, Crouzon syndrome, Pfeiffer syndrome, Jackson-Weiss syndrome)	*FGFR2*	0.0042637	-1.98	-2.02
B2.i2	fibroblast growth factor receptor 2 (bacteria-expressed kinase, keratinocyte growth factor receptor, craniofacial dysostosis 1, Crouzon syndrome, Pfeiffer syndrome, Jackson-Weiss syndrome)	*FGFR2*	0.0193394	-2.06	
D10.a4	BOTL0100008_E02		0.0362718	-2.27	-2.00
D10.e9	BOTL0100008_E02		0.0236260	-1.73	

BOTL-5 coordinates	Feature description	Gene symbol	*P*-value	Fold-change	Mean fold-change
A5.i4	nuclear receptor co-repressor 1	*NCOR1*	0.0335313	-1.74	-2.00
B9.d8	nuclear receptor co-repressor 1	*NCOR1*	0.0005404	-2.01	
B9.h9	nuclear receptor co-repressor 1	*NCOR1*	0.0002447	-2.24	
C12.b8	transformer-2 alpha	*TRA2A*	0.0023524	-1.90	-1.96
C12.g2	transformer-2 alpha	*TRA2A*	0.0034953	-2.01	
A4.d8	conserved helix-loop-helix ubiquitous kinase	*CHUK*	0.0250975	-1.74	-1.93
A4.h9	conserved helix-loop-helix ubiquitous kinase	*CHUK*	0.0203143	-2.12	
C5.e1	macrophage migration inhibitory factor (glycosylation-inhibiting factor)	*MIF*	0.0218428	-1.74	-1.91
C5.i3	macrophage migration inhibitory factor (glycosylation-inhibiting factor)	*MIF*	0.0061871	-2.07	
C4.e2	protein_kinase_C,_alpha_binding_protein		0.0381032	-1.92	-1.89
C4.i4	protein_kinase_C,_alpha_binding_protein		0.0498164	-1.86	
C11.b7	BOTL0100009_H06		0.0357204	-1.84	-1.88
C11.g1	BOTL0100009_H06		0.0166329	-1.92	
B1.d8	Rho guanine nucleotide exchange factor (GEF) 1	*ARHGEF1*	0.0090614	-1.74	-1.88
B1.h9	Rho guanine nucleotide exchange factor (GEF) 1	*ARHGEF1*	0.0081270	-2.01	
D3.e3	MAP kinase interacting serine/threonine kinase 1	*MKNK1*	0.0311134	-1.97	-1.87
D3.i5	MAP kinase interacting serine/threonine kinase 1	*MKNK1*	0.0294027	-1.77	
A6.b8	SET binding factor 1	*SBF1*	0.0072729	-1.77	-1.84
A6.g2	SET binding factor 1	*SBF1*	0.0043798	-1.90	

BOTL-5 coordinates	Feature description	Gene symbol	*P*-value	Fold-change	Mean fold-change
B8.e1	RAS p21 protein activator 3	*RASA3*	0.0195049	-1.83	-1.83
B8.i3	RAS p21 protein activator 3	*RASA3*	0.0292993	-1.82	
D1.e6	serum response factor (c-fos serum response element-binding transcription factor)	*SRF*	0.0108486	-1.91	-1.83
D1.i7	serum response factor (c-fos serum response element-binding transcription factor)	*SRF*	0.0465517	-1.74	
C9.b7	eukaryotic translation initiation factor 5	*EIF5*	0.0110766	-1.88	-1.81
C9.g1	eukaryotic translation initiation factor 5	*EIF5*	0.0346893	-1.74	
A7.a5	uncoupling protein 2 (mitochondrial, proton carrier)	*UCP2*	0.0005476	-1.83	-1.80
A7.f1	uncoupling protein 2 (mitochondrial, proton carrier)	*UCP2*	0.0029412	-1.78	
C4.b9	triggering receptor expressed on myeloid cells-1	*TREM-1*	0.0454033	-1.83	-1.77
C4.g3	triggering receptor expressed on myeloid cells-1	*TREM-1*	0.0385547	-1.71	
B2.a3	chromosome 12 open reading frame 35	*C12orf35*	0.0387424	-1.62	-1.77
B2.e8	chromosome 12 open reading frame 35	*C12orf35*	0.0309394	-1.92	
D6.e1	CASP8 and FADD-like apoptosis regulator	*CFLAR*	0.0273795	-1.72	-1.71
D6.i3	CASP8 and FADD-like apoptosis regulator	*CFLAR*	0.0042129	-1.71	
A11.a4	major histocompatibility complex, class II, DR alpha	*HLA-DRA*	0.0031203	-1.62	-1.69
A11.e9	major histocompatibility complex, class II, DR alpha	*HLA-DRA*	0.0023432	-1.75	
B4.b3	glutathione synthetase	*GSS*	0.0196135	-1.65	-1.68
B4.f7	glutathione synthetase	*GSS*	0.0078180	-1.71	
C11.b3	bromodomain containing 2	*BRD2*	0.0313898	-1.80	-1.68

BOTL-5 coordinates	Feature description	Gene symbol	*P*-value	Fold-change	Mean fold-change
C11.f7	bromodomain containing 2	*BRD2*	0.0170426	-1.55	
A4.a4	splicing factor proline/glutamine-rich (polypyrimidine tract binding protein associated)	*SFPQ*	0.0036285	-1.62	-1.67
A4.e9	splicing factor proline/glutamine-rich (polypyrimidine tract binding protein associated)	*SFPQ*	0.0004477	-1.73	
A9.a8	BOTL0100003XA07R		0.0225451	-1.58	-1.65
A9.f5	BOTL0100003XA07R		0.0061795	-1.72	
B8.b7	prohibitin 2	*PHB2*	0.0161357	-1.34	-1.65
B8.g1	prohibitin 2	*PHB2*	0.0193058	-1.47	
D5.e1	prohibitin 2	*PHB2*	0.0020928	-1.93	
D5.i3	prohibitin 2	*PHB2*	0.0073849	-1.86	
B9.a3	BOTL0100003XG08R		0.0046742	-1.71	-1.63
B9.e8	BOTL0100003XG08R		0.0210765	-1.56	
B4.b5	BOTL0100008_H02		0.0023660	-1.66	-1.63
B4.f8	BOTL0100008_H02		0.0041364	-1.60	
A11.a6	similar to Lymphocyte antigen Ly-6E precursor (Retinoic acid-induced gene E protein) (RIG-E) (Thymic shared antigen 1) (TSA-1) (Stem cell antigen 2) (SCA-2)	*MGC140297*	0.0431527	-1.62	-1.63
A11.f2	similar to Lymphocyte antigen Ly-6E precursor (Retinoic acid-induced gene E protein) (RIG-E) (Thymic shared antigen 1) (TSA-1) (Stem cell antigen 2) (SCA-2)	*MGC140297*	0.0410107	-1.64	
D2.d8	TANK-binding kinase 1	*TBK1*	0.0004030	-1.75	-1.63
D2.h9	TANK-binding kinase 1	*TBK1*	0.0096930	-1.50	
C8.a4	Wiskott-Aldrich syndrome (eczema-thrombocytopenia)	*WAS*	0.0153904	-1.64	-1.60

BOTL-5 coordinates	Feature description	Gene symbol	*P*-value	Fold-change	Mean fold-change
C8.e9	Wiskott-Aldrich syndrome (eczema-thrombocytopenia)	*WAS*	0.0265945	-1.57	
D4.b7	BOTL0100013_F01		0.0001301	-1.50	-1.59
D4.g1	BOTL0100013_F01		0.0021400	-1.67	
A1.a7	BOTL0100002XD04R		0.0006579	-1.51	-1.58
A1.f3	BOTL0100002XD04R		0.0070696	-1.64	
C2.d8	protein kinase C, beta 1	*PRKCB1*	0.0479493	-1.58	-1.54
C2.h9	protein kinase C, beta 1	*PRKCB1*	0.0210312	-1.50	
C4.a4	mannosyl (alpha-1,6-)-glycoprotein beta-1,2-N-acetylglucosaminyltransferase	*MGAT2*	0.0034894	-1.53	-1.52
C4.e9	mannosyl (alpha-1,6-)-glycoprotein beta-1,2-N-acetylglucosaminyltransferase	*MGAT2*	0.0039099	-1.50	
A10.a8	nurim (nuclear envelope membrane protein)	*NRM*	0.0135772	-2.67	-1.50
A10.f5	nurim (nuclear envelope membrane protein)	*NRM*	0.0009069	-3.47	
C8.f8	nurim (nuclear envelope membrane protein)	*NRM*	0.0062870	1.66	
C8.e2	SMAD family member 7	*SMAD7*	0.0031160	-1.54	-1.49
C8.i4	SMAD family member 7	*SMAD7*	0.0126812	-1.45	
A3.c5	immediate early response 5	*IER5*	0.0179898	-1.27	-1.49
A3.g8	immediate early response 5	*IER5*	0.0385969	-1.27	
B12.a5	immediate early response 5	*IER5*	0.0039860	-1.53	
B12.f1	immediate early response 5	*IER5*	0.0359498	-1.38	
B2.b9	immediate early response 5	*IER5*	0.0102766	-1.70	

BOTL-5 coordinates	Feature description	Gene symbol	*P*-value	Fold-change	Mean fold-change
B2.g3	immediate early response 5	*IER5*	0.0129934	-1.77	
D2.a3	Cbp/p300-interacting transactivator, with Glu/Asp-rich carboxy-terminal domain, 2	*CITED2*	0.0170796	-1.47	-1.48
D2.e8	Cbp/p300-interacting transactivator, with Glu/Asp-rich carboxy-terminal domain, 2	*CITED2*	0.0320792	-1.48	
A6.b9	hypothetical LOC506974	*LOC506974*	0.0013024	-1.46	-1.47
A6.g3	hypothetical LOC506974	*LOC506974*	0.0028593	-1.49	
D8.b9	Ran GTPase activating protein 1	*RANGAP1*	0.0082155	-1.41	-1.43
D8.g3	Ran GTPase activating protein 1	*RANGAP1*	0.0023614	-1.45	
D6.b7	serine dehydratase-like	*SDSL*	0.0445689	-1.41	-1.43
D6.g1	serine dehydratase-like	*SDSL*	0.0464412	-1.45	
C11.c1	BTG family, member 3	*BTG3*	0.0013132	-1.46	-1.41
C11.g4	BTG family, member 3	*BTG3*	0.0135524	-1.36	
B3.e4	phosphoinositide-3-kinase, regulatory subunit 5, p101	*PIK3R5*	0.0358222	-1.41	-1.40
B3.i6	phosphoinositide-3-kinase, regulatory subunit 5, p101	*PIK3R5*	0.0450732	-1.40	
A5.b7	actin-bundling protein with BAIAP2 homology	*ABBA-1*	0.0258944	-1.37	-1.40
A5.g1	actin-bundling protein with BAIAP2 homology	*ABBA-1*	0.0078472	-1.43	
C9.e2	Mdm2, transformed 3T3 cell double minute 2, p53 binding protein (mouse)	*MDM2*	0.0037452	-1.31	-1.39
C9.i4	Mdm2, transformed 3T3 cell double minute 2, p53 binding protein (mouse)	*MDM2*	0.0090137	-1.48	
D4.b6	Fanconi anemia, complementation group A	*FANCA*	0.0166410	-1.37	-1.38
D4.f9	Fanconi anemia, complementation group A	*FANCA*	0.0066791	-1.40	

BOTL-5 coordinates	Feature description	Gene symbol	*P*-value	Fold-change	Mean fold-change
A4.c1	zinc finger protein 250	*ZNF250*	0.0132725	-1.31	-1.37
A4.g4	zinc finger protein 250	*ZNF250*	0.0078097	-1.43	
A4.b9	ELL associated factor 1	*EAF1*	0.0039927	-1.34	-1.30
A4.g3	ELL associated factor 1	*EAF1*	0.0091441	-1.26	
A6.a4	nudix (nucleoside diphosphate linked moiety X)-type motif 8	*NUDT8*	0.0024655	-1.16	-1.17
A6.e9	nudix (nucleoside diphosphate linked moiety X)-type motif 8	*NUDT8*	0.0332678	-1.17	
A3.g4	protein tyrosine phosphatase, receptor type, C-associated protein	*PTPRCAP*	0.0363632	-1.44	-0.38
B5.a3	protein tyrosine phosphatase, receptor type, C-associated protein	*PTPRCAP*	0.0266610	1.54	
D2.b8	protein tyrosine phosphatase, receptor type, C-associated protein	*PTPRCAP*	0.0400525	-1.24	
B9.a5	zinc finger, DHHC-type containing 7	*ZDHHC7*	0.0411976	1.38	-0.06
B9.f1	zinc finger, DHHC-type containing 7	*ZDHHC7*	0.0331515	1.45	
D3.a5	zinc finger, DHHC-type containing 7	*ZDHHC7*	0.0309508	-1.49	
D3.f1	zinc finger, DHHC-type containing 7	*ZDHHC7*	0.0275724	-1.59	
A6.f7	poly (ADP-ribose) polymerase family, member 10	*PARP10*	0.0095845	-1.34	0.09
C10.a4	poly (ADP-ribose) polymerase family, member 10	*PARP10*	0.0290333	1.52	
C11.b5	similar to PRIP-interacting protein PIPMT	*LOC511392*	0.0312712	1.30	1.34
C11.f8	similar to PRIP-interacting protein PIPMT	*LOC511392*	0.0149358	1.37	
C6.b9	transcription elongation factor A (SII)-like 4	*TCEAL4*	0.0100420	1.33	1.35
C6.g3	transcription elongation factor A (SII)-like 4	*TCEAL4*	0.0370660	1.37	

BOTL-5 coordinates	Feature description	Gene symbol	*P*-value	Fold-change	Mean fold-change
D9.d7	caspase 10, apoptosis-related cysteine peptidase	*CASP10*	0.0017394	1.38	1.40
D9.h7	caspase 10, apoptosis-related cysteine peptidase	*CASP10*	0.0149277	1.42	
B11.a3	hydroxysteroid (17-beta) dehydrogenase 4	*HSD17B4*	0.0333190	1.35	1.40
B11.e8	hydroxysteroid (17-beta) dehydrogenase 4	*HSD17B4*	0.0093751	1.45	
C12.b9	BOTL0100012_G03		0.0378284	1.53	1.42
C12.g3	BOTL0100012_G03		0.0181028	1.30	
D10.b7	methyltransferase like 5	*METTL5*	0.0273635	1.44	1.43
D10.g1	methyltransferase like 5	*METTL5*	0.0395425	1.42	
C6.d9	protein phosphatase 2, regulatory subunit B', beta isoform	*PPP2R5B*	0.0132696	1.38	1.43
C6.i2	protein phosphatase 2, regulatory subunit B', beta isoform	*PPP2R5B*	0.0009699	1.49	
C3.b6	BOTL0100009_D12		0.0161245	1.45	1.44
C3.f9	BOTL0100009_D12		0.0058036	1.44	
B10.a4	transducin (beta)-like 1X-linked	*TBL1X*	0.0489704	1.44	1.49
B10.e9	transducin (beta)-like 1X-linked	*TBL1X*	0.0176459	1.54	
D10.b3	malignant T cell amplified sequence 1	*MCTS1*	0.0430731	1.47	1.51
D10.f7	malignant T cell amplified sequence 1	*MCTS1*	0.0219207	1.54	
A4.e3	28S_ribosomal_RNA_gene		0.0004790	1.58	1.52
A4.i5	28S_ribosomal_RNA_gene		0.0032932	1.46	
C10.c5	similar to leukocyte differentiation antigen CD84	*LOC510910*	0.0375443	1.50	1.53

BOTL-5 coordinates	Feature description	Gene symbol	*P*-value	Fold-change	Mean fold-change
C10.g8	similar to leukocyte differentiation antigen CD84	*LOC510910*	0.0165416	1.56	
C7.a8	osteoglycin	*OGN*	0.0039958	1.58	1.53
C7.f5	osteoglycin	*OGN*	0.0043193	1.48	
D5.c2	BOTL0100011_F08		0.0033658	1.53	1.56
D5.g5	BOTL0100011_F08		0.0023427	1.59	
D10.a3	tyrosine 3-monooxygenase/tryptophan 5-monooxygenase activation protein, gamma polypeptide	*YWHAG*	0.0471314	1.43	1.57
D10.e8	tyrosine 3-monooxygenase/tryptophan 5-monooxygenase activation protein, gamma polypeptide	*YWHAG*	0.0048779	1.72	
A6.e3	AXL receptor tyrosine kinase	*AXL*	0.0141779	1.65	1.59
A6.i5	AXL receptor tyrosine kinase	*AXL*	0.0290260	1.52	
C4.i8	AXL receptor tyrosine kinase	*AXL*	0.0085442	1.59	
A9.c1	BOTL0100010_D08		0.0330704	1.58	1.59
A9.g4	BOTL0100010_D08		0.0046436	1.59	
C9.c2	BOTL0100010_D12		0.0299158	1.79	1.61
C9.g5	BOTL0100010_D12		0.0404501	1.42	
D8.d7	platelet-derived growth factor beta polypeptide (simian sarcoma viral (v-sis) oncogene homolog)	*PDGFB*	0.0321179	1.57	1.61
D8.h7	platelet-derived growth factor beta polypeptide (simian sarcoma viral (v-sis) oncogene homolog)	*PDGFB*	0.0158677	1.65	
C12.c5	BOTL0100013_D12		0.0326116	1.77	1.61
C12.g8	BOTL0100013_D12		0.0015900	1.46	
B9.a7	transportin 2 (importin 3, karyopherin beta 2b)	*TNPO2*	0.0063557	1.35	1.63

BOTL-5 coordinates	Feature description	Gene symbol	*P*-value	Fold-change	Mean fold-change
B9.f3	transportin 2 (importin 3, karyopherin beta 2b)	*TNPO2*	0.0017477	1.91	
B9.b9	BOTL0100011_C03		0.0050056	1.71	1.66
B9.g3	BOTL0100011_C03		0.0224630	1.60	
B5.c1	BOTL0100011_F05		0.0132287	1.64	1.68
B5.g4	BOTL0100011_F05		0.0091696	1.72	
A12.b7	BOTL0100012_F09		0.0029204	1.68	1.68
A12.g1	BOTL0100012_F09		0.0143107	1.69	
B8.d7	platelet-derived growth factor alpha polypeptide	*PDGFA*	0.0190129	1.73	1.70
B8.h7	platelet-derived growth factor alpha polypeptide	*PDGFA*	0.0262092	1.68	
C10.c8	proprotein convertase subtilisin/kexin type 6	*PCSK6*	0.0240355	1.73	1.73
C10.h2	proprotein convertase subtilisin/kexin type 6	*PCSK6*	0.0087853	1.73	
B4.d7	myeloproliferative leukemia virus oncogene	*MPL*	0.0115917	1.71	1.74
B4.h7	myeloproliferative leukemia virus oncogene	*MPL*	0.0068954	1.77	
D8.d5	endothelial cell growth factor 1 (platelet-derived)	*ECGF1*	0.0229457	1.75	1.77
D8.h6	endothelial cell growth factor 1 (platelet-derived)	*ECGF1*	0.0173043	1.79	
A12.c4	HD domain containing 2	*HDDC2*	0.0010459	1.93	1.82
A12.g6	HD domain containing 2	*HDDC2*	0.0027764	1.71	
C6.c7	melanin-concentrating hormone receptor 1	*MCHR1*	0.0487027	1.90	1.84
C6.h1	melanin-concentrating hormone receptor 1	*MCHR1*	0.0174139	1.79	

BOTL-5 coordinates	Feature description	Gene symbol	*P*-value	Fold-change	Mean fold-change
B11.e3	G protein-coupled receptor 98	*GPR98*	0.0003538	2.08	2.07
B11.i5	G protein-coupled receptor 98	*GPR98*	0.0061638	2.07	
